# Edge‐Rich Landscapes Promote Genetic Diversity While Arable Land Restricts It: Landscape‐Genomic Patterns Across Three Insect Species in an Agriculture‐Dominated Grassland Mosaic

**DOI:** 10.1111/eva.70326

**Published:** 2026-10-01

**Authors:** Christopher Wild, Belinda Kahnt, Sebastian Hopfenmüller, Bilyana Stoykova Wild, Cinja Schwarz, Florian Fumy, Robert J. Paxton, Thomas Fartmann, Panagiotis Theodorou

**Affiliations:** ^1^ General Zoology, Institute for Biology Martin Luther University Halle‐Wittenberg Halle (Saale) Germany; ^2^ Naturkundemuseum Erfurt Erfurt Germany; ^3^ German Centre for Integrative Biodiversity Research (iDiv) Halle‐Jena‐Leipzig Leipzig Germany; ^4^ Stiftung KulturLandschaft Günztal Ottobeuren Germany; ^5^ Division of Biodiversity and Landscape Ecology, Department of Biology Osnabrück University Osnabrück Germany

**Keywords:** agricultural landscapes, conservation management, genetic diversity, habitat connectivity, landscape genetics, population structure

## Abstract

Habitat loss and agricultural intensification are major drivers of insect decline, yet their consequences for intraspecific genetic diversity remain poorly understood. Here, we used landscape genomics to compare genome‐wide diversity, population differentiation and spatial patterns of gene flow in two butterfly species (*Polyommatus icarus*, *Melanargia galathea*) and one bush‐cricket species (*Conocephalus fuscus*) sampled across an agriculturally dominated landscape in southern Germany. The study region consists of a heterogeneous mosaic of extensively managed open grasslands maintained through conservation initiatives and embedded within intensively managed grasslands, arable fields and forest patches. High‐resolution genomic data combined with broad geographic sampling revealed pronounced interspecific differences. The highly mobile, widespread and generalist *P. icarus* exhibited the highest diversity and weakest population genetic structure, the moderately widespread *M. galathea* showed intermediate levels, whereas the comparatively less mobile orthopteran *C. fuscus* displayed the lowest genetic diversity and strongest population subdivision. Across the three species, genetic diversity was positively associated with edge density and showed a tendency to decline with increasing arable cover, indicating that landscape configuration influences genetic variation more strongly than habitat area alone. Effective migration surfaces showed broadly shared spatial patterns, with lower effective migration in intensively farmed areas and higher effective migration in grassland‐rich parts of the study region. Together, our results highlight that landscape heterogeneity and species‐specific ecological characteristics jointly determine genetic resilience in insects. Our findings support conserving networks of structurally diverse, well‐connected grasslands and reducing homogeneous arable matrices to help maintain genetic diversity and evolutionary potential in insect communities.

## 1 Introduction

The ongoing degradation of natural habitats caused by anthropogenic activities has led to unprecedented biodiversity loss, undermining ecosystem functioning and threatening services vital to human well‐being (Sharma and Birman 2024). In response, conservation efforts increasingly emphasise the establishment of ecological networks designed to maintain or restore connectivity across fragmented landscapes (Langhammer et al. 2024). In Europe, initiatives such as the Natura 2000 network and regional projects like the Biotopverbund Günztal in Bavaria aim to promote biodiversity by preserving or re‐establishing ecological continuity (Evans 2012; Hopfenmüller et al. 2015). However, the effectiveness of such conservation strategies remains insufficiently evaluated, particularly from a population‐genetic perspective, where substantial knowledge gaps persist (Coates et al. 2018; Starnes et al. 2021). Evaluating these genetic outcomes is important because demographic patterns alone may not reveal changes in genetic diversity, gene flow or population isolation that can affect the long‐term persistence of populations (Höglund 2009).

Insects, despite representing the majority of terrestrial biodiversity and playing essential roles in ecosystems (e.g., pollination, nutrient cycling and food‐web dynamics), remain under‐represented in conservation planning (Cardoso et al. 2011; Chowdhury, Zalucki, et al. 2023; Goulson 2019). This gap is particularly concerning given mounting evidence for widespread declines in insect abundance, richness and biomass across many regions (Cardoso et al. 2020; Fartmann et al. 2021; Hallmann et al. 2017; Seibold et al. 2019; Van Klink et al. 2020, 2024; Wagner 2020), including declines in formerly common species (Brower et al. 2012; Van Klink et al. 2024). These trends are driven by interacting pressures such as habitat loss, fragmentation, agricultural intensification and climate change (Wagner et al. 2021). Despite these threats, insect conservation often relies on incidental protection through umbrella effects or general habitat protection rather than targeted strategies (Morán‐López et al. 2020; Samways et al. 2020). Consequently, the capacity of protected or extensively managed landscapes to maintain the genetic health of insect populations remains insufficiently tested (Chowdhury, Jennions, et al. 2023). Genomic approaches offer considerable potential to address this knowledge gap by quantifying genetic diversity, population connectivity and responses to anthropogenic landscape change, yet they remain rarely incorporated into insect conservation practice (Sucháčková Bartoňová et al. 2023).

Anthropogenic land use nowadays dominates most temperate landscapes (Ellis et al. 2021), and can strongly affect insect population genetics by reducing habitat availability, increasing isolation among populations and fragmenting previously continuous habitats (Gaublomme et al. 2013; Hill et al. 2006; Nolen et al. 2026). Reduced dispersal and gene flow can intensify genetic drift and inbreeding, ultimately increasing extinction risk (Jacobson et al. 2019; Jha 2015; Schleimer and Frantz 2025). Among land use pressures, agricultural expansion and intensification are among the most pervasive, generally exerting negative effects on insect populations (Brown and Paxton 2009; Vanbergen and The Insect Pollinators Initiative 2013). Intensification often simplifies landscapes, removes semi‐natural structures and disrupts resource continuity (Arancibia et al. 2020; Benton et al. 2003; Landis 2017; Raven and Wagner 2021; Shi et al. 2024), processes that can accelerate the loss of intraspecific genetic diversity (Allendorf et al. 2022; Andersen et al. 2004). These genetic consequences may depend not only on landscape composition, such as the amount of suitable habitat or arable land, but also on its spatial configuration (Balkenhol 2015). Landscape configuration can influence functional connectivity through the spatial arrangement of habitat patches, edges and potential movement pathways. Structurally heterogeneous landscapes containing habitat edges and semi‐natural elements may therefore facilitate dispersal and gene flow, whereas homogeneous agricultural matrices may increase population isolation. Consequently, both landscape composition and configuration can shape the maintenance of genetic diversity in agricultural landscapes. Evidence of genetic erosion associated with anthropogenic land use change has been documented in diverse insect taxa, including burnet moths, bush‐crickets, butterflies, bees and aquatic insects (Crossley et al. 2023; Della Rocca et al. 2025; Habel et al. 2012; Nolen et al. 2026; Suni and Hernandez 2023). Yet it remains unclear whether existing conservation tools, particularly protected areas, can effectively mitigate these genetic losses.

Protected areas are often assumed to support higher levels of genetic diversity and connectivity (Martinez et al. 2002; Pérez‐Ruzafa et al. 2006), but empirical evidence for insects remains limited and inconsistent (Thompson et al. 2021). For example, González‐Castellano et al. (2023) found no detectable genetic benefits of protection for the Spanish moon moth, *Graellsia isabellae* (Lepidoptera: Saturniidae), highlighting the need to evaluate when and for which species protected areas effectively sustain population genetic health. Addressing this question requires considering species‐specific ecological traits. Insects vary widely in dispersal ability, habitat specialisation, voltinism, body size and resource use, traits that strongly influence both genetic diversity and population structure (López‐Uribe et al. 2019; Webster et al. 2023). Highly mobile habitat generalists typically maintain larger effective population sizes and higher gene flow, resulting in greater genetic diversity and weaker population structure (Packer et al. 2005). In contrast, habitat specialists or species with limited dispersal often occur in small, fragmented populations that are more susceptible to genetic erosion and may therefore benefit most from well‐designed conservation networks (Packer et al. 2005). Consequently, the same landscape may differ in its permeability among species, potentially resulting in contrasting population‐genetic responses to agricultural intensification and habitat configuration. Comparative studies across species with contrasting ecological traits are therefore essential for evaluating the effectiveness of landscape‐scale conservation measures.

Here, we investigate how human land use practices and the resulting landscape composition and configuration are associated with insect genetic diversity, population structure and spatial patterns of effective migration in a predominantly intensively managed agricultural landscape interspersed with areas of extensive management and conservation activity. We focus on three insect species, two Lepidoptera (*Polyommatus icarus* and *Melanargia galathea*) and one Orthoptera species (*Conocephalus fuscus*), that differ in dispersal capacity, habitat specialisation and ecological requirements. The study was conducted in a heterogeneous agricultural landscape comprising arable fields, intensively managed grasslands, forest patches and smaller areas of extensively managed open grasslands within the Biotopverbund Günztal. This system provides an opportunity to examine whether landscape characteristics associated with extensive management and structural heterogeneity are related to population‐genomic patterns across species with contrasting ecologies.

Specifically, we tested the following three hypotheses: (1) Genetic diversity will increase with the amount of extensively managed grassland and with landscape heterogeneity, reflected by greater edge density, because these landscapes may provide more habitat and facilitate movement among populations. Conversely, increasing arable cover and fragmentation of extensively managed grasslands are expected to be associated with reduced genetic diversity. (2) Species‐specific ecological traits influence patterns of genetic diversity and population structure. Species with limited dispersal are expected to show lower genetic diversity and stronger population subdivision than more mobile species, resulting in the strongest structure in *C. fuscus* , intermediate structure in *M. galathea* and the weakest structure in *P. icarus* . Genetic differentiation among populations is also expected to increase with geographic distance, consistent with isolation by distance. (3) Despite differences in dispersal ability, all species are expected to show broadly similar spatial patterns of effective migration, with higher connectivity in the structurally heterogeneous and extensively managed southern part of the study region and lower connectivity in agriculturally intensified northern and eastern areas.

## 2 Material and Methods

### 2.1 Study Area and Sampling Sites

Our study was conducted in the Günz Valley in southwestern Bavaria, Germany (Figure 1). The study area spans approximately 77 km in a north–south direction. Although the valley's initial geomorphology was shaped by Pleistocene glacial processes, anthropogenic influences, particularly agricultural intensification and the long‐standing dominance of dairy farming, have been the primary drivers of landscape change over the last few centuries. Within this strongly anthropogenically shaped landscape, the ‘Kulturlandschaft Günztal’ foundation and partner organisations have implemented conservation measures aimed at establishing extensively managed meadows. These meadows are typically characterised by two to three mowing events per year and the exclusion of fertilisers and mulching. Across the valley, a pronounced management gradient is evident: some sectors contain a relatively high density of extensively managed open grassland patches, partly reflecting a long history of coordinated conservation efforts, whereas the majority of the valley remains dominated by intensive agricultural use (Figure 1).

**FIGURE 1:**
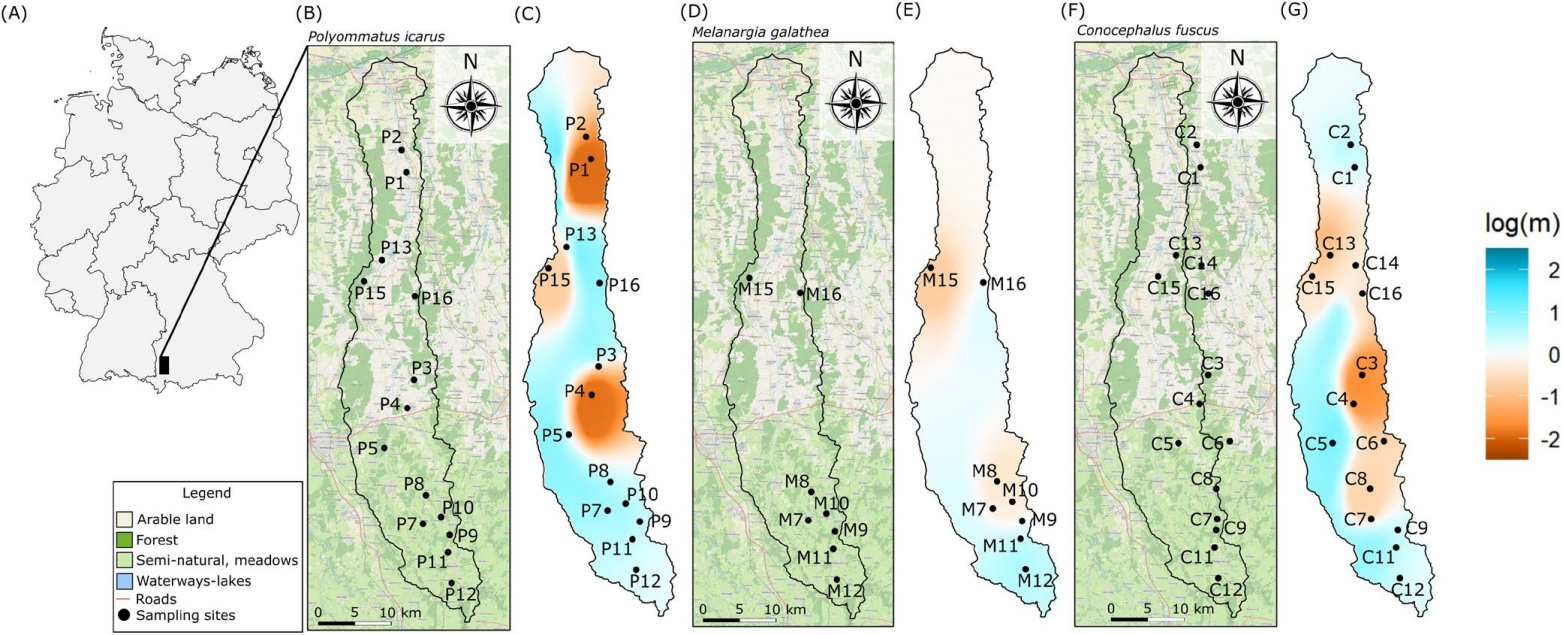
Location of the Günz Valley within Germany (A). Also shown are the sampling sites (black circles) and effective migration surfaces for *Polyommatus icarus* (B, C), *Melanargia galathea* (D, E) and *Conocephalus fuscus* (F, G). Orange coloured areas correspond to areas with less effective migration than expected under an isolation‐by‐distance model (negative log migration rates, log(*m*)), whereas blue coloured areas indicate higher effective migration than expected (positive log migration rates). The base map was obtained from OpenStreetMap, the national borders of Germany were sourced from a freely accessible repository (https://github.com/isellsoap/deutschlandGeoJSON). Data are licensed by the OpenStreetMap Foundation (OSMF) under the Open Database License (ODbL) (https://opendatacommons.org/licenses/odbl/).

To capture variation in landscape composition and configuration across the study region, we surveyed 16 sampling sites, although not all species occurred at every site (Figure 1; Tables S1–S3). Sampling sites for *P. icarus* and *M. galathea* were located in extensively managed meadows. For *C. fuscus* , some sampling locations were situated along edges or in the immediate vicinity of the extensively managed meadows that were selected for the other species (see Table S3 for exact GPS coordinates). Thus, sampling was concentrated within or near extensively managed grasslands while encompassing substantial variation in the composition and configuration of the surrounding landscape. To ensure statistical independence among populations, sites were separated by a minimum distance of 2 km (Figure 1).

### 2.2 Study Species and Sampling

We selected two Lepidoptera species, *P. icarus* (common blue butterfly) and *M. galathea* (marbled white butterfly), and one Orthoptera species, *C. fuscus* (long‐winged conehead bush‐cricket), as focal taxa. These species were chosen because they are widespread in Central European grassland ecosystems and exhibit contrasting ecological traits, including dispersal ability, habitat specialisation and life‐history strategies (Bellmann et al. 2019; Tolman and Lewington 1997).

*Polyommatus icarus* is a multivoltine butterfly widely distributed across Europe and commonly found in open habitats, including extensively managed meadows, semi‐natural grasslands and roadside verges (Emmet and Heath 1989; Rivest and Kharouba 2021; Tolman and Lewington 1997). Its larvae feed primarily on leguminous host plants such as *Coronilla* spp., *Lotus corniculatus* and *Medicago lupulina* (Tolman and Lewington 1997), whereas adults consume nectar from a variety of Fabaceae, including *L. corniculatus* . The species exhibits moderate to high dispersal ability, with mark–release–recapture studies reporting movements ranging from several hundred metres to maximum recorded movements of up to 1457 m (Gutiérrez et al. 2001; Rivest and Kharouba 2021).

*Melanargia galathea* is a univoltine grassland specialist typically occurring in nutrient‐poor, semi‐dry meadows and calcareous grasslands (Terzer et al. 2024; Tolman and Lewington 1997; Vandewoestijne et al. 2004). Within the study area, the species is generally less abundant than *P. icarus* (Fumy et al. 2023; Voith et al. 2016a; Tolman and Lewington 1997). Its larvae feed on a range of grasses, including *Brachypodium* and *Poa* spp., whereas adults feed on a wide range of flowering plants, including *Centaurea*, *Knautia*, *Scabiosa* and *Cirsium* spp. (Tolman and Lewington 1997). Studies indicate high within‐patch mobility with extensive mixing of individuals within habitat patches, whereas movements between habitat patches appear comparatively limited, with observed dispersal distances of up to approximately 600 m (Habel et al. 2010).

*Conocephalus fuscus* is a long‐winged bush‐cricket commonly found in moist grasslands, wet meadows, riparian zones, fallow land and ruderal habitats (Bellmann et al. 2019). It is phytophagous and partly omnivorous, feeding on grasses as well as small invertebrates (Bellmann et al. 2019). The species uses acoustic signalling for mate location, a behaviour that promotes active movement within structurally complex vegetation (Avci and Taylan 2023; Löffler et al. 2019; Aleksanov 2025). *Conocephalus fuscus* is fully winged, indicating the ability to fly and disperse between habitat patches. However, to our knowledge, no quantitative estimates of dispersal distances have been published for this species. Based on its behavioural ecology and the generally limited capacity of orthopterans for sustained flight, *C. fuscus* is expected to exhibit lower effective dispersal across the landscape than the two butterfly species examined here.

Whenever possible, a minimum of 10 individuals per species was collected at each sampling site. Insects were captured using hand nets during species‐specific sampling periods: *P. icarus* from May to August 2020 (*n* = 155; sampled at 14 sites), *M. galathea* from July to August 2020 (*n* = 77; sampled at 8 sites) and *C. fuscus* from July to September 2020 (*n* = 153; sampled at 15 sites) (Tables S1–S3). Species identification was carried out in the field based on morphological characteristics. Sex was recorded based on morphological characteristics for *P. icarus* and *C. fuscus* . Corresponding information was not available for *M. galathea* (Tables S1–S3). All individuals were preserved in 96% ethanol in 5 mL Eppendorf tubes and subsequently stored at −20°C for long‐term preservation.

### 2.3 DNA Extraction, Restriction‐Site Associated DNA (RAD) Sequencing and Sequence Assembly

To generate single nucleotide polymorphisms (SNP) markers across the genome for the study species, high‐molecular‐weight DNA was extracted from thoracic muscle tissue for *P. icarus* and *M. galathea* and from a saltatorial leg for *C. fuscus*. DNA extraction was performed using either the DNeasy Blood & Tissue Kit (Qiagen) or a CTAB‐based protocol with proteinase K digestion, followed by chloroform:isoamyl alcohol extraction (24:1). DNA quality was assessed using an Epoch spectrophotometer (BioTek, Winooski, USA) and agarose gel electrophoresis, while DNA quantity was quantified with a Qubit 3.0 fluorometer (Thermo Fisher Scientific, Waltham, MA, USA). DNA extracts were standardised to a final volume of 30 μL at a final concentration of 17 ± 3 ng/μL before sequencing.

Samples were submitted to SNPsaurus for Nextera‐tagmented reductively‐amplified DNA (NextRAD) sequencing, a reduced‐representation sequencing approach that combines Nextera tagmentation with selective PCR amplification to target a reproducible subset of genomic loci for high‐throughput SNP genotyping. Libraries were sequenced on an Illumina NovaSeq 6000 platform (University of Oregon) using one lane of 122 bp single‐end reads. Sequencing output per sample averaged 3.25 million reads for *P. icarus* (SD = 1.14; mean depth of 34×, SD = 12), 3.31 million reads for *M. galathea* (SD = 1.00; mean depth of 22×, SD = 4.94) and 4.91 million reads for *C. fuscus* (SD = 0.74; mean depth of 16×, SD = 2.37) (Tables S4–S6).

Quality filtering and adapter trimming of raw sequencing reads were performed using *BBDuk* from the *BBMap* suite (Bushnell 2014). Reads were processed to remove adapters, short sequences and low‐quality bases using the following parameters: minlen = 100, ktrim = r, *k* = 17, mink = 8, hdist = 1, qtrim = rl, trimq = 10 and ordered = t. Cleaned reads were assembled and analysed using *ipyrad* v.0.9.102 (Eaton and Overcast 2020). For *P. icarus* and *M. galathea*, reads were mapped to their respective reference genomes ( *P. icarus* : ilPolIcar1.1, GCA_937595015.1; *M. galathea*: ilMelGala2.1, GCA_920104075.1). Because no reference genome was available for *C. fuscus* , RAD loci for this species were assembled de novo.

Within *ipyrad*, sequences were clustered using a sequence‐similarity threshold of 0.90 (clust_threshold = 0.90). Reads containing more than five low‐quality or ambiguous bases were excluded (max_low_qual_bases = 5; Phred *Q*‐score offset = 33). A minimum read depth of six was required for both statistical and majority‐rule consensus base calling (mindepth_statistical = 6; mindepth_majrule = 6) and the maximum within‐sample cluster depth was set to 1000 (maxdepth = 1000). Consensus sequences were restricted to a maximum of two alleles (max_alleles_consens = 2), a maximum proportion of 0.05 undetermined bases (max_Ns_consens = 0.05) and a maximum proportion of 0.05 heterozygous sites (max_Hs_consens = 0.05). During final locus filtering, loci were required to occur in at least four individuals (min_samples_locus = 4). Loci containing more than 20% SNPs (max_SNPs_locus = 0.2), more than eight indels (max_Indels_locus = 8) or more than 50% shared heterozygous sites (max_shared_Hs_locus = 0.5) were excluded; the latter criterion is designed to identify potentially paralogous loci. The resulting *ipyrad* assemblies contained 14,104,825 loci for 155 individuals of *P. icarus* , 8,074,859 loci for 77 individuals of *M. galathea* and 20,608,081 loci for 153 individuals of *C. fuscus* .

### 2.4 Genomic Dataset Preparation and Filtering

Following assembly and initial locus filtering in *ipyrad*, individuals with > 50% missing genotype data were removed from all subsequent analyses using the ‐‐missing‐indv function in *VCFtools* v. 0.1.16 (Danecek et al. 2011). This resulted in the removal of 17 individuals of *P. icarus* (*n* = 138/155), one individual of *M. galathea* (*n* = 76/77) and six individuals of *C. fuscus* (*n* = 147/153). Using the remaining individuals, we generated two datasets for downstream analyses: (i) a dataset containing polymorphic SNPs for analyses of genetic differentiation, population structure and effective migration; and (ii) a dataset containing both polymorphic and monomorphic sites for estimates of genome‐wide genetic diversity. The number of sites retained at each filtering step for each species is provided in Table S7.

For *P. icarus* and *M. galathea*, for which reference genomes were available, sites located on the sex chromosomes were excluded from the *ipyrad* output prior to the preparation of downstream analytical datasets to avoid potential biases in estimates of genetic diversity, differentiation and population structure. For comparison, all downstream analyses for both butterfly species were repeated using datasets in which sex‐linked sites were retained, with the corresponding results presented in the Supporting Information. Results were highly consistent between datasets with and without sex‐linked sites, and their inclusion did not alter the statistical interpretation of any analysis.

For analyses of genetic differentiation and population structure, SNPs were filtered using *VCFtools* v. 0.1.16 (Danecek et al. 2011). SNPs were filtered using the following parameters: ‐‐max‐missing 0.85, ‐‐maf 0.05 and ‐‐remove‐indels. After filtering, and after exclusion of sex‐linked SNPs for the two butterfly species, 6642 SNPs were retained for *P. icarus* , 22,493 SNPs for *M. galathea* and 13,160 SNPs for *C. fuscus* . To account for linkage disequilibrium (LD), SNPs were pruned using *PLINK* v1.9 (Purcell et al. 2007) with ‐‐indep 50 5 0.5, which removes markers in high LD (*r* ^2^ > 0.5). To avoid potential biases caused by family structure, we tested for highly related individuals using ‐‐relateness2 in *VCFtools* (Manichaikul et al. 2010). Relatedness values between 0.177 and 0.354 indicate second‐degree relatives. No highly related individuals were detected. As an additional screen for potentially paralogous loci, loci with observed heterozygosity (*H*_o_) > 0.6 were excluded. No loci exceeded this threshold in any of the three species and, consequently, this filtering step did not reduce the number of retained loci. After filtering and LD pruning, the final dataset used for analyses of genetic differentiation and population structure contained 5038 SNPs for 138 individuals of *P. icarus* , 15,561 SNPs for 76 individuals of *M. galathea* and 9678 SNPs for 147 individuals of *C. fuscus* .

To estimate genome‐wide genetic diversity, we used a second dataset including both polymorphic and monomorphic sites. This approach is less sensitive to variation in sample size and allows more reliable comparisons of genetic diversity across species than estimates based solely on polymorphic loci (Schmidt et al. 2021; Sopniewski and Catullo 2024). For this dataset, *ipyrad* outputs were filtered to retain all SNPs and monomorphic loci that passed quality filtering. Filtering criteria required that at least 85% of individuals had genotype data for a given locus (‐‐max‐missing 0.85) and indels were removed (‐‐remove‐indels). As an additional screen for potentially paralogous sites, sites with observed heterozygosity (*H*_o_) > 0.6 were excluded. No sites exceeded this threshold in any of the three species, and therefore this filtering step did not reduce the number of retained sites. The final datasets used to estimate genome‐wide genetic diversity comprised 501,542 polymorphic and monomorphic loci for 138 individuals of *P. icarus* , 1,149,641 loci for 76 individuals of *M. galathea* and 1,081,825 loci for 147 individuals of *C. fuscus* .

### 2.5 Genetic Diversity, Differentiation and Population Genetic Structure

Genome‐wide genetic diversity was quantified as unbiased, sample size–corrected expected heterozygosity (*uH*_EXP_), using the R package *dartR* v. 2.9.7 (Gruber et al. 2018). Estimates were calculated from both polymorphic and monomorphic loci to ensure comparability across populations and species. Pairwise genetic differentiation (*F*_ST_) was estimated using *StAMPP* (Pembleton et al. 2013) based on biallelic polymorphic SNPs. Confidence intervals for *F*_ST_ estimates were obtained using bootstrap resampling (*n* = 1000). Isolation by distance (IBD) was assessed using Mantel tests implemented in the R package *ade4* v. 1.7‐22 (Dray and Dufour 2007), correlating linearised *F*_ST_ values [*F*_ST_/(1 − *F*_ST_)] with log‐transformed geographic distances (km) between population pairs for each species.

Population genetic structure was inferred by estimating individual ancestry coefficients using *sNMF* implemented in the R package *LEA* v. 3.14 (Frichot and François 2015). This method applies matrix factorisation with sparse regularisation to cluster individuals based on multilocus genotype data. For *M. galathea*, values of *K* = 1–9 were tested, whereas *K* = 1–15 was evaluated for *P. icarus* and *C. fuscus* , reflecting their broader geographic sampling coverage. For each value of *K*, 10 replicate runs were performed, and the most likely number of clusters was determined using the lowest cross‐entropy criterion. As an additional approach to evaluate population structure, we conducted a discriminant analysis of principal components (DAPC) using the R package *adegenet* v. 2.10 (Jombart 2008; Jombart and Ahmed 2011). The optimal number of retained principal components was determined using *α*‐score optimisation.

To assess whether multilocus genetic variation was associated with sex, we additionally analysed *P. icarus* and *C. fuscus* , for which sex information was available. Principal component analyses (PCAs) were conducted separately for each species using the filtered, LD‐pruned SNP dataset, and individuals were visualised by population and sex to assess potential sex‐specific clustering. Differences in multilocus genetic variation between males and females were additionally tested using permutational multivariate analysis of variance (PERMANOVA) with the *adonis2* function in the R package *vegan* v. 2.7‐5 (Oksanen et al. 2026). The effect of sex was tested using 999 permutations restricted within populations to account for underlying population genetic structure. Corresponding PCA ordinations are presented in Figures S1 and S2. Consistent with this pattern, PERMANOVA with permutations restricted within populations detected no significant association between sex and multilocus genetic variation in *P. icarus* (*F* = 0.779, *R* ^2^ = 0.005, *p* = 1) or *C. fuscus* (*F* = 0.952, *R* ^2^ = 0.006, *p* = 0.985).

### 2.6 Effective Migration Surfaces (EEMS)

To investigate spatial patterns of gene flow and identify regions of restricted or elevated migration, we used the Estimated Effective Migration Surfaces (EEMS) framework (Petkova 2025; Petkova et al. 2016). EEMS models the relationship between genetic and geographic distances by estimating spatial variation in effective migration rates across a user‐defined landscape. EEMS analyses were run using 300 demes for *P. icarus* , 150 demes for *M. galathea* and 300 demes for *C. fuscus* . The differing numbers of demes reflect differences in spatial sampling extent among species: species sampled more densely and across a broader geographic range ( *P. icarus* and *C. fuscus* ) allow finer spatial resolution, whereas more limited sampling (*M. galathea*) requires a coarser mesh to avoid overparameterisation and unstable estimates. For each species, the Markov chain Monte Carlo (MCMC) algorithm was run for 6,000,000 iterations, including a burn‐in of 3,000,000 iterations, with thinning every 999 steps. These settings were determined through preliminary runs to ensure adequate mixing and effective sampling of the posterior distribution. Convergence of each chain was assessed visually using posterior log‐likelihood trace plots generated with the *reemsplots2* R package (Petkova 2025; Petkova et al. 2016).

### 2.7 Landscape Composition and Configuration

Landscape composition and configuration were analysed using QGIS v.3.16.3, incorporating land cover data from Geofabrik GmbH (OpenStreetMap‐based data; http://www.geofabrik.de/). To improve the accuracy of the Geofabrik land cover map, anonymised IACS (Integrated Administration and Control System) data provided by the Bavarian state government were integrated. These data provided a more detailed representation of land use surrounding the sampling sites. Remaining gaps in land cover classification were manually verified using satellite imagery from Google Earth Pro v.7.3.6.9750.

Landscape composition was quantified as the proportional cover of four major land cover types: arable land, extensively managed open grassland, intensively managed meadows and forest. Landscape metrics were calculated in R version 4.2.2 (R Core Team 2024). Extensively managed open grassland included hay meadows, litter meadows, low‐intensity pastures, green fallows, annual flowering fallows and all meadows known to be managed extensively (two to three mowings per year without fertilisation or mulching) under the supervision of the Kulturlandschaft Günztal Foundation or associated stakeholders. Intensively managed meadows were characterised by a higher input intensity (e.g., fertilisation), more frequent mowing (five to seven mowings per year) and reduced structural heterogeneity compared with extensively managed open grassland.

Landscape configuration was quantified using two metrics. First, edge density was calculated as the total length of land cover boundaries divided by the total landscape area (m^2^) within a given spatial scale. Edges represent boundaries between adjacent land cover patches (e.g., grassland–forest, grassland–arable field or between similar land cover types such as extensively managed grasslands). Edge density was used as a proxy for structural landscape heterogeneity and potential habitat connectivity because it reflects the density of ecotones in the landscape (Fahrig 2017; Martin et al. 2019). Second, we calculated patch density as a metric of habitat fragmentation for extensively managed open grasslands by dividing the number of habitat patches by their total area within each buffer radius, providing a measure of habitat subdivision independent of overall habitat extent (Fahrig 2017; Theodorou, Herbst, et al. 2020). Because landscape variables may influence genetic diversity at different spatial scales, all landscape metrics were calculated within buffer radii of 500, 700 and 1000 m around the centroid (midpoint) of each sampled meadow. To identify the spatial scale with the most explanatory power for each predictor, landscape metrics calculated at each scale were correlated with *uH*_EXP_ for each species. The spatial scale with the highest correlation coefficient was retained for subsequent statistical analyses (Table S8).

### 2.8 Statistical Analyses

All statistical analyses were conducted in R v.4.4.2 (R Core Team 2024). To test for differences in genome‐wide genetic diversity among species while accounting for variation in sample size, we fitted a linear model (LM) with *uH*_EXP_ as the response variable and species identity and the number of individuals per population (*n*) as predictors. Estimated marginal means (EMMs) for each species were obtained using the *emmeans* R package (Lenth and Piaskowski 2025), and pairwise species comparisons were conducted using Tukey‐adjusted contrasts. To test whether *F*_ST_ differed among species while accounting for geographic distance, we first fitted a LM with *F*_ST_ as the response variable and species identity and geographic distance as predictors. Because inspection of model residuals indicated heteroscedasticity among species, we estimated species‐specific residual variances and used their inverse as weights in a Weighted Least Squares (WLS) model. EMMs were then calculated for each species, and pairwise contrasts were performed to evaluate differences in *F*_ST_.

To investigate the effects of landscape composition and configuration on *uH*_EXP_, we fitted species‐specific LMs. Predictor variables included the proportional cover of arable land, extensively managed open grassland, intensively managed meadows and forest, as well as edge density and the fragmentation index for extensively managed open grassland. Prior to modelling, all predictors were standardised (z‐transformed) to a mean of zero and a standard deviation of one. Multicollinearity among predictors was assessed using variance inflation factors (VIF), applying a threshold of VIF < 5 (James et al. 2013). No evidence of problematic multicollinearity was detected. Given the relatively large number of predictors, we fitted a global model for each species and performed automated model selection using the *dredge* function in the *MuMIn* package (Bartoń 2010). To reduce the risk of overfitting, candidate models were restricted to a maximum of three predictors for *P. icarus* and *C. fuscus* and two predictors for *M. galathea*. Models within ΔAICc < 2 of the top‐ranked model were subsequently averaged to obtain model‐averaged parameter estimates.

Model assumptions, including linearity, normality, homogeneity of variance and residual distributions, were assessed using the *DHARMa* R package (Hartig 2024). To evaluate potential spatial autocorrelation in model residuals, we calculated Moran's *I* using the *ape* R package (Gittleman and Kot 1990). No significant spatial autocorrelation was detected in any of the final models (*p* > 0.05).

## 3 Results

### 3.1 Genome‐Wide Genetic Diversity

Genome‐wide genetic diversity measured as *uH*_EXP_ ranged from 0.0074 to 0.0077 for *P. icarus* , 0.0063–0.0067 for *M. galathea* and 0.0039–0.0044 for *C. fuscus* (Tables S1–S3). Genetic diversity differed significantly between species (*uH*_EXP_: LM; EMM contrasts: *p* < 0.001; Figure 2). *Conocephalus fuscus* exhibited the lowest genetic diversity, followed by *M. galathea*, whereas *P. icarus* showed the highest genetic diversity (Figure 2A).

**FIGURE 2:**
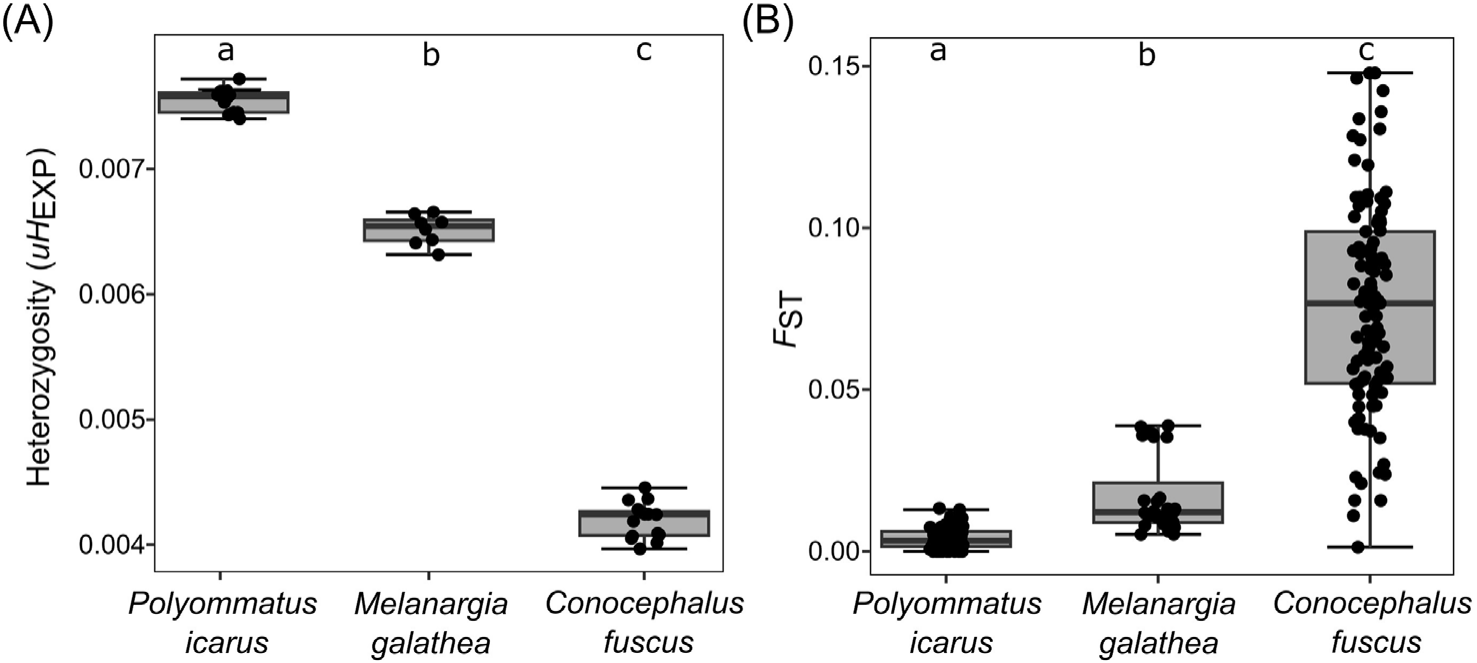
(A) Genome‐wide genetic diversity, measured as unbiased expected heterozygosity (*uH*_EXP_), and (B) pairwise population genetic differentiation, measured as *F*_ST_, for the three insect species studied. Boxes represent the interquartile range, with the horizontal line indicating the median. Different letters indicate significant differences between species.

The effects of landscape composition and configuration on genome‐wide genetic diversity were assessed separately for each species using LMs. For *P. icarus* , the best‐supported model (ΔAICc < 2) included edge density within a 500 m radius and the proportion of arable cover within a 700 m radius (Table 1). Edge density was positively and significantly associated with genetic diversity (*uH*_EXP_: LM; *z* = 2.356, *p* = 0.038; Figure 3A), whereas arable cover showed a significant negative association with genetic diversity (*uH*_EXP_: LM; *z* = −2.859, *p* = 0.016; Figure 3B). For *M. galathea*, two models fell within ΔAICc < 2 (Table 1), with the null model receiving the strongest support. The second model included edge density at a 500 m radius, which showed a positive but non‐significant relationship with genetic diversity (*uH*_EXP_: LM; *z* = 2.02, *p* = 0.089; Figure 3C). For completeness, we also present the relationship between arable cover and genetic diversity in *M. galathea* (*uH*_EXP_: LM; *z* = −1.152, *p* = 0.293; Figure 3D), although arable cover did not appear in any supported models. For *C. fuscus* , two models fell within ΔAICc < 2 (Table 1). Both included edge density within a 500 m radius, which showed a strong and significant positive association with genetic diversity (*uH*_EXP_: LM; *z* = 2.904, *p* = 0.003; Figure 4A). The second model additionally included the proportion of arable cover within a 1000 m radius, which exhibited a negative but non‐significant relationship with genetic diversity (*uH*_EXP_: LM; *z* = −1.129, *p* = 0.258; Figure 4B).

**TABLE 1:** Model selection statistics and model‐averaged coefficients (conditional average) for the genome‐wide genetic diversity (*uH*_EXP_) of *Polyommatus icarus*, *Melanargia galathea* and *Conocephalus fuscus* .

Response	Model	AICc	ΔAICc	Weight	Predictor	*z* value	*p*	*R* ^2^
*P. icarus* *uH*_EXP_	~Edge density (500 m radius) + proportion of arable cover (700 m radius)	−221.53	0.00	0.29	Edge density (500 m radius)	2.356	0.038*	0.17
					Proportion of arable cover (700 m radius)	−2.859	0.016*	0.30
*M. galathea uH*_EXP_	~NULL	−116.35	0.00	0.480	Edge density (500 m)	2.02	0.089^ns^	0.305
	~Edge density (500 m radius)	−114.90	1.45	0.230				
*C. fuscus uH*_EXP_	~Edge density (500 m radius)	−224.62	0.00	0.350	Edge density (500 m radius)	2.904	0.003**	0.407
	~ Edge density (500 m) + Proportion of arable cover (1000 m radius)	−222.66	1.97	0.130	Proportion of arable cover (1000 m radius)	−1.129	0.258^ns^	0.145

*Note:* Significance levels: ns = *p* > 0.05; *p < 0.05; **p < 0.01.

**FIGURE 3:**
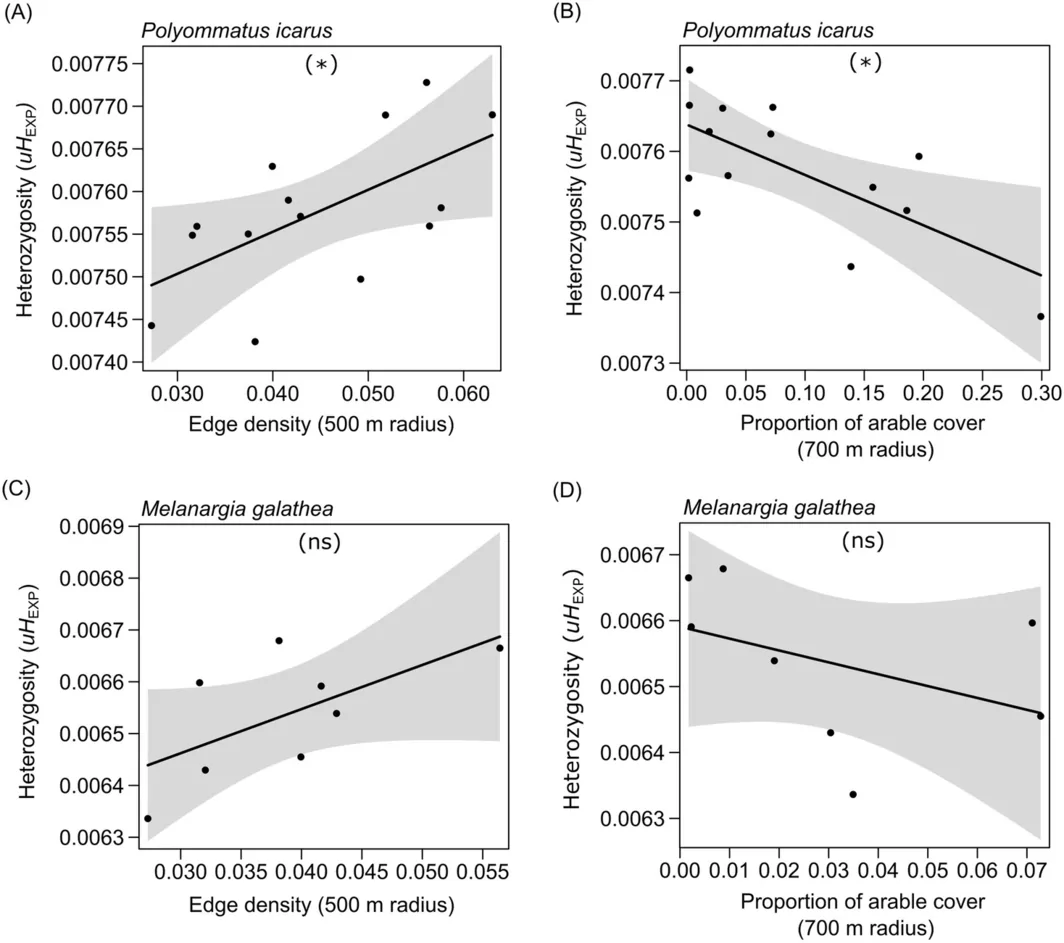
Relationships between genome‐wide genetic diversity (*uH*_EXP_) and edge density (500 m radius) and arable cover for *Polyommatus icarus* (A, B) and *M. galathea* (C, D). Solid lines represent predicted relationships from linear models and shaded areas indicate 95% confidence intervals. Points represent partial residuals. Significance levels: ns = *p* > 0.05; *p < 0.05.

**FIGURE 4:**
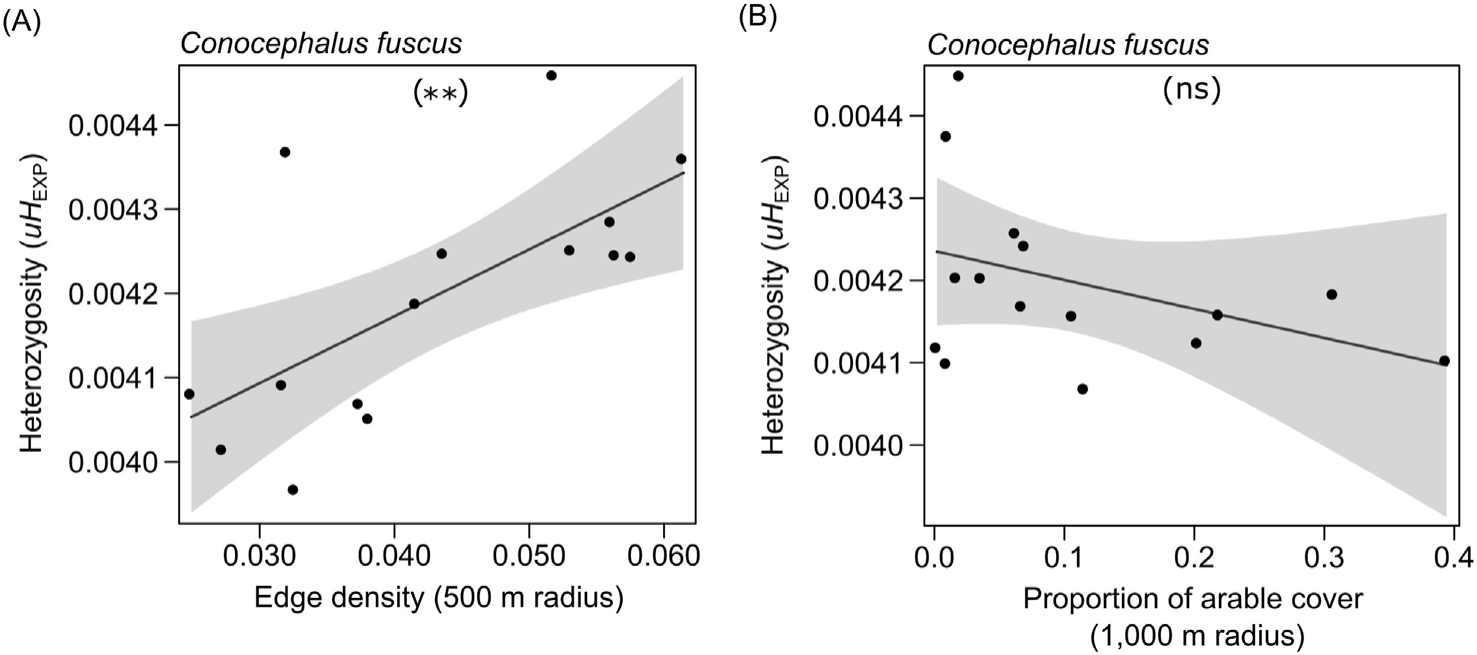
Relationships between genome‐wide genetic diversity (*uH*_EXP_) in *Conocephalus fuscus* and (A) edge density (500 m radius) and (B) the proportion of arable cover (1000 m radius). Solid lines represent predicted relationships from linear models and shaded areas indicate 95% confidence intervals. Points represent partial residuals. Significance levels: ns = *p* > 0.05; **p < 0.01.

Retaining sex‐linked sites in *P. icarus* and *M. galathea* did not alter the overall patterns of genome‐wide genetic diversity or its relationships with landscape variables (Figures S3 and S4).

### 3.2 Genetic Divergence, Population Structure and Effective Migration Surfaces

Genetic divergence between populations was low but significantly different from zero in all species ( *P. icarus* : mean *F*_ST_ = 0.004, 95% CI = 0.001–0.007; *M. galathea*: mean *F*_ST_ = 0.017, 95% CI = 0.015–0.019; *C. fuscus* : mean *F*_ST_ = 0.069, 95% CI = 0.064–0.073; Tables S9–S11). Species differed significantly in their levels of genetic differentiation. *Polyommatus icarus* exhibited the lowest pairwise *F*_ST_ values, followed by *M. galathea*, whereas *C. fuscus* showed the highest genetic differentiation (Weighted Least Squares model; EMM contrasts: *p* < 0.001; Figure 2B).

Mantel tests revealed no significant correlation between genetic and geographic distances for *P. icarus* (*r* = −0.083, *p* > 0.05; Figure S5) and *C. fuscus* (*r* = 0.07, *p* > 0.05; Figure S5), indicating no evidence of IBD in these species. In contrast, *M. galathea* showed a significant positive correlation between population genetic differentiation and geographic distances (*r* = 0.623, *p* = 0.013; Figure S5), suggesting IBD.

Discriminant analysis of principal components, optimised using *α*‐score selection, retained 24 principal components for *P. icarus* , 13 for *M. galathea* and 15 for *C. fuscus* . Together with the *sNMF* cross‐entropy analyses, the results revealed clear interspecific differences in population genetic structure (Figure 5). *Polyommatus icarus* showed very weak genetic structuring, with substantial overlap among populations in the DAPC and no distinct clusters, consistent with its low *F*_ST_ values (Figure 5A). In contrast, *M. galathea* exhibited more pronounced structure, with several populations displaying partial or clear separation from others. Nevertheless, *sNMF* supported *K* = 1, indicating little evidence for discrete ancestral population structure despite the differentiation observed among sampling populations (Figure 5B). *Conocephalus fuscus* exhibited the strongest population structure, with several sampling populations clearly separated in the DAPC and limited overlap among them (Figure 5C). This pattern was also evident in *sNMF*, for which cross‐entropy was lowest at *K* = 6, supporting substantially stronger population subdivision than in either butterfly species.

**FIGURE 5:**
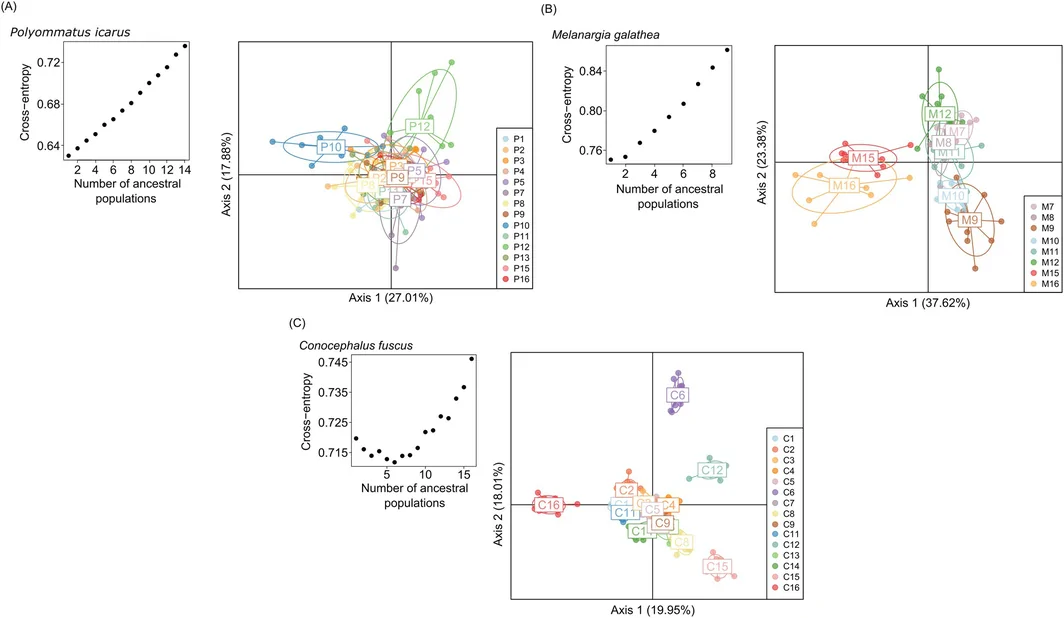
Genetic structure analyses based on discriminant analysis of principal components (DAPC) and cross‐entropy values for the range of genetic clusters (*K*) tested with sNMF for (A) *Polyommatus icarus*, (B) *Melanargia galathea* and (C) *Conocephalus fuscus* . The value of *K* with the lowest cross‐entropy score was taken as the most likely number of population clusters for each species. In the DAPC plots, each population is shown in a different colour. Axes represent the first two discriminant functions, with labels indicating the proportion of variance explained. Ellipses indicate 95% confidence limits around population centroids.

EEMS analyses revealed spatial variation in effective migration rates across the Günz Valley for all three species (Figure 1C,E,G). In *P. icarus* , elevated migration was detected mainly in the southern part of the valley and along a corridor extending from the southwest towards the central northeast and northwest, whereas reduced migration occurred in the south‐central–eastern region and in two northern areas (Figure 1C). In *M. galathea*, elevated migration was inferred in the southern sector and along a southwest–northeast corridor (Figure 1E). For *C. fuscus* , elevated effective migration was observed in the north, central, south‐western, and south regions, whereas reduced migration was identified in an area extending from the southeast to the northeast and northwest (Figure 1G).

Analyses retaining sex‐linked SNPs in *P. icarus* and *M. galathea* yielded results consistent with the autosomal analyses across genetic differentiation, isolation by distance, population structure and effective migration (Figures S3 and S6–S8).

## 4 Discussion

Understanding how landscape composition and configuration shape genetic diversity and connectivity is essential for assessing the resilience of insect populations in human‐modified grassland systems, particularly in intensively managed landscapes with local conservation initiatives such as the Biotopverbund Günztal. By analysing two butterflies ( *P. icarus* and *M. galathea*) and one bush‐cricket ( *C. fuscus* ) across the same mosaic of differently managed meadows and arable fields, we show that co‐occurring species differ markedly in their genetic patterns yet also exhibit notable similarities. Both *P. icarus* and *C. fuscus* exhibited higher genetic diversity in edge‐rich areas, while increasing arable cover was associated with lower genetic diversity in *P. icarus* and showed a similar, although non‐significant, tendency in *C. fuscus* . *Melanargia galathea* exhibited effects in the same direction but with weaker, non‐significant relationships. EEMS analyses also revealed some broadly similar spatial patterns of high and low connectivity across the three taxa, suggesting that the configuration of the Günz Valley landscape influences gene flow in a broadly consistent manner despite differences in species' ecology.

Because sampling sites consisted of extensively managed open grasslands, local habitat conditions were broadly comparable and represented suitable habitat for the focal species. Thus, suitable habitat availability formed the baseline condition for species occurrence, whereas variation in the surrounding landscape primarily shaped patterns of genetic connectivity and genetic diversity among populations. This hierarchical perspective is consistent with landscape genetic theory, in which suitable habitat enables population persistence, whereas landscape structure governs dispersal and gene flow, thereby influencing patterns of genetic diversity among populations (Holderegger and Wagner 2008; Manel et al. 2003). Together, these results suggest that landscape heterogeneity and agricultural simplification leave shared genetic signatures across species, even though the strength and detectability of these effects vary depending on species ecology and sampling resolution. Below, we evaluate these patterns in the context of our hypotheses and the ecological characteristics of each species.

### 4.1 Interspecific Differences in Genetic Diversity and Genetic Divergence

The three species showed a clear gradient in genome‐wide genetic diversity and population differentiation: *P. icarus* exhibited the highest diversity and weakest genetic divergence, *M. galathea* intermediate values and *C. fuscus* the lowest diversity and strongest differentiation. These differences likely reflect a combination of regional abundance, distribution patterns and intrinsic life‐history traits. *Polyommatus icarus* has a broad Palearctic range, maintains dense regional populations and is the most common of the three taxa in Bavaria, whereas *C. fuscus* occupies patchier wet‐meadow habitats and is only moderately common (Detzel 1998; Voith et al. 2016b). Differences in abundance and population continuity may translate into variation in effective population size (*N*_e_), potentially contributing to the observed hierarchy in genetic diversity.

The stronger differentiation and lower genetic diversity observed in *C. fuscus* should nevertheless be interpreted with some caution because RAD loci for this species were assembled de novo, whereas reads for *P. icarus* and *M. galathea* were mapped to reference genomes. De novo clustering and reference‐based mapping can differ in the loci and alleles they recover, potentially affecting estimates of heterozygosity, allele frequencies and population differentiation. Although large numbers of sites were retained for all three species and stringent depth, missingness and heterozygosity filters were applied, we cannot completely exclude an influence of assembly strategy on the observed interspecific differences.

Despite this methodological difference, the observed interspecific gradient is consistent with broader taxonomic and ecological patterns. Comparative studies indicate that life‐history and ecological traits, rather than geographic range alone, are major determinants of genetic diversity (Ellegren and Galtier 2016; Romiguier et al. 2014). Butterflies generally disperse further, exploit more continuous habitat networks, and form larger metapopulations than many orthopterans, and consequently tend to exhibit higher genetic diversity and weaker structure (Reinhardt et al. 2005). Grasshoppers and bush‐crickets, particularly those restricted to wet or semi‐natural meadows, often occur in smaller and more isolated populations, making them more susceptible to genetic drift and stronger population subdivision (Gardiner 2010; Streiff et al. 2006; Weyer et al. 2012).

Life‐history traits of the focal species further reinforce these patterns: *P. icarus* is multivoltine and has a long flight period, whereas *M. galathea* and *C. fuscus* are univoltine, and *M. galathea* has a relatively short adult activity window. Together, these ecological and demographic differences provide a coherent explanation for the pronounced interspecific gradient in genetic diversity and population differentiation observed in this study. Future work including additional insect taxa could help clarify how specific life‐history traits shape genomic diversity patterns across insect groups.

Potential effects of sex and sex‐linked variation are also unlikely to account for the observed interspecific differences. The inclusion of sex‐linked sites did not influence the overall genetic patterns in the two butterfly species, while no evidence of sex‐associated genome‐wide genetic differentiation was found in *P. icarus* or *C. fuscus* . Although sex‐linked loci could not be specifically identified and excluded in *C. fuscus* because a suitable reference genome was unavailable, the absence of detectable genomic differences between males and females suggests that sex‐associated variation is unlikely to explain the stronger population differentiation observed in this species.

### 4.2 Effects of Landscape Composition and Configuration on Genetic Diversity

Species differed in how strongly landscape features were associated with genome‐wide genetic diversity, yet common directional patterns emerged. All three taxa showed higher heterozygosity in edge‐rich areas, although the strength and statistical support for this relationship varied across species. A positive association between edge density and genome‐wide heterozygosity has also recently been reported in the solitary bee *Andrena florea* (Baltz et al. 2026), suggesting that this relationship may extend across insect taxa and landscape contexts. Ecotones, or transitions between habitat types, can support local populations and facilitate short‐distance movement by providing microclimatic refugia, foraging resources or linear movement pathways (Brebner et al. 2021; Mallinoff et al. 2025; Theodorou et al. 2022; Theodorou, Radzevičiūtė, et al. 2020). Because larger or better‐connected populations tend to maintain higher genetic diversity (Frankham et al. 2002), the positive association with edge density may reflect greater demographic stability and/or functional connectivity in structurally heterogeneous landscapes. The occurrence of similar directional relationships in two butterflies and one bush‐cricket suggests that ecotone‐rich, structurally heterogeneous landscapes can promote genetic diversity in insects with different ecological traits.

Arable cover showed the opposite pattern: genetic diversity declined with increasing arable dominance in all three species, although the relationship was statistically significant only for *P. icarus* . Intensive agriculture reduces habitat continuity and resource availability and can constrain dispersal, thereby lowering effective population sizes and increasing the influence of genetic drift (Clarke et al. 2024; Habel et al. 2019; Hemberger et al. 2023; Jauker et al. 2009; Waples 2025). Although the effect of arable land cover was statistically strongest in *P. icarus* , the consistently negative trend across species suggests a general vulnerability of grassland insects to agricultural homogenisation. These results likely reflect the historical land use intensification and current landscape configuration of the Günz Valley, where large contiguous arable areas, especially in the northern part of the valley, may hinder movement and reduce demographic stability.

The weaker statistical signal in *M. galathea* warrants cautious interpretation. Although this species showed the same directional responses as the other two taxa, positive with edge density and negative with arable cover, neither relationship was statistically significant. The most likely explanation is limited analytical power; *M. galathea* had a smaller sample size and occurred within a much narrower landscape gradient, particularly with respect to arable cover, which varied only weakly among its sampling sites. Restricted environmental variation can mask landscape–genetic effects even when biological responses are present (Balkenhol 2015). Thus, limited sample size and environmental variation may have reduced our ability to detect landscape associations in *M. galathea*. However, we cannot distinguish this possibility from genuinely weaker landscape effects in this species. Additional sampling across a broader landscape gradient would be required to differentiate between these explanations.

Extensively managed open grassland did not appear in any of the best‐supported models, indicating that, within the range of conditions represented in our survey area, its explanatory power for genome‐wide genetic diversity was weaker than that of edge density or arable cover. Importantly, this should not be interpreted as evidence that extensive management is unimportant. All sampling sites were located within extensively managed open grasslands, providing a common local habitat condition, while the amount of extensively managed grassland and the composition and configuration of the surrounding landscape varied among sites. Our results therefore indicate that variation in the amount of extensive grassland surrounding sampling sites did not explain detectable among‐population variation in genetic diversity within the landscape gradient studied.

From an ecological perspective, extensively managed grasslands are known to support higher abundance and species richness of Lepidoptera and Orthoptera by reducing mowing frequency, limiting fertilisation and maintaining structural heterogeneity (Chisté et al. 2016; Fumy and Fartmann 2023; Guariento et al. 2023; Hannappel and Fischer 2020; Jerrentrup et al. 2014; Schwarz et al. 2023). Higher local abundance is expected to increase effective population size and buffer populations against genetic drift (Frankham et al. 2002), thereby contributing to the maintenance of genetic diversity. Such benefits would not necessarily generate detectable differences among populations in our study, however, because all sampled populations occupied extensively managed grasslands. This interpretation is consistent with the limited empirical evidence showing that extensive management or protected‐area status does not necessarily translate directly into higher genetic diversity in insects. For example, González‐Castellano et al. (2023) found no genetic differences between *Graellsia isabellae* populations inside versus outside protected areas. Genetic responses to contemporary conservation management may also depend on population connectivity, effective population size and historical land use, and may occur only after demographic and evolutionary time lags. Consequently, contemporary landscape–genetic patterns may integrate both current landscape structure and historical processes.

From a conservation perspective, our findings highlight the importance of maintaining or restoring fine‐scale habitat transitions within agricultural landscapes. Increasing edge density, particularly at boundaries between extensively managed grasslands, semi‐natural habitats and riparian zones, may help sustain genetic diversity even in species with moderate mobility. Because structurally heterogeneous landscapes benefited all three species, promoting ecotone‐rich mosaics represents a promising multi‐species conservation strategy for the Biotopverbund Günztal. Conversely, high and spatially aggregated arable cover may pose a genetic risk by reducing local population sizes and limiting functional connectivity. Maintaining extensively managed grasslands while increasing structural heterogeneity and incorporating additional semi‐natural landscape elements may therefore help support the long‐term genetic resilience of grassland insect populations in the Günz Valley.

### 4.3 Genetic Divergence, Isolation by Distance and Effective Migration Surfaces

Despite the modest geographic extent of the study area, pairwise *F*_ST_ values were significantly greater than zero in all species, indicating some degree of restricted gene flow and self‐recruitment. Even *P. icarus* , the most mobile species, exhibited low but detectable population differentiation, suggesting subtle demographic processes and incomplete genetic homogenisation across the landscape. *Conocephalus fuscus*, with its patchy distribution and lower mobility, showed the strongest differentiation, consistent with its ecological constraints (Detzel 1998).

Patterns of IBD differed notably among species. *Melanargia galathea* showed a clear positive correlation between genetic and geographic distance, indicating that dispersal is spatially constrained and that gene flow declines with increasing distance. In contrast, neither *P. icarus* nor *C. fuscus* exhibited significant IBD. For *P. icarus* , the absence of IBD is consistent with its capacity for relatively widespread dispersal (Cowley et al. 2001; Schmitt et al. 2003; Tolman and Lewington 1997), which likely homogenises genetic variation across the study area. The lack of IBD in *C. fuscus* likely reflects a different mechanism: rather than high mobility, populations may be shaped by localised recruitment and patch‐specific demographic histories. Such dynamics can generate heterogeneous *F*_ST_ values unrelated to geographic distance, particularly when unsuitable matrix habitats restrict movement in spatially heterogeneous ways (Frankham et al. 2002).

Spatial patterns of effective migration inferred from EEMS further illustrate how landscape structure shapes connectivity in all three species across the Günz Valley. Despite their contrasting dispersal abilities, the species shared broadly similar migration landscapes. Elevated effective migration was consistently inferred in the southern Günz Valley, which is characterised by high habitat heterogeneity, a long history of extensive meadow management, lower agricultural intensification and smaller proportions of arable land. In contrast, reduced effective migration occurred primarily in the central–eastern and northern parts of the valley, regions dominated by intensive agriculture and lower structural diversity. These congruent migration patterns across species suggest that certain areas of the landscape systematically facilitate or impede movement, largely independent of species‐specific traits. Notably, the southern sector of the Günz Valley appears to function as a multi‐species connectivity zone, whereas intensively cultivated northern and eastern sections represent recurrent barriers to gene flow.

From a conservation perspective, these results highlight two priority actions: (1) the protection and maintenance of the southern connectivity corridor, where gene flow remains high across all taxa; and (2) the restoration of habitat heterogeneity in northern and eastern agricultural areas, where reduced migration suggests that current land use constrains functional connectivity. Overall, the combined *F*_ST_, IBD and EEMS results reveal differing levels of genetic differentiation among species but a remarkably consistent spatial pattern of connectivity barriers and corridors, underscoring the central role of landscape configuration in shaping multi‐species gene flow in fragmented agricultural landscapes.

## 5 Conclusion

This study shows that landscape composition and configuration are associated with genetic diversity and connectivity in insects inhabiting an agriculturally dominated landscape. Across species, genome‐wide genetic diversity was consistently higher in edge‐rich landscapes and tended to decline with increasing arable cover. Spatial patterns of effective migration also showed similarities across taxa, with comparatively high migration repeatedly observed in the more heterogeneous southern Günz Valley and reduced migration in several more intensively cultivated parts of the landscape. Together, these findings suggest that structurally heterogeneous agricultural landscapes containing extensively managed grasslands and abundant habitat transitions may contribute to maintaining genetic diversity and connectivity across insect species with contrasting ecological traits. Maintaining such heterogeneous landscapes while limiting agricultural homogenisation may therefore help support the long‐term genetic resilience of insect populations in fragmented grassland systems.

## Funding

This work was supported by the Bundesamt für Naturschutz (BfN), the Bundesministerium für Umwelt, Naturschutz, nukleare Sicherheit und Verbraucherschutz (BMU) and the Bayerischer Naturschutzfonds—Stiftung des öffentlichen Rechts (BMU Grant No. 3519685C18).

## Disclosure

Benefit Sharing Statement: Benefits from this research accrue from the sharing of our data and results on public databases as described above.

## Conflicts of Interest

The authors declare no conflicts of interest.

## Supporting information


**Figure S1:** Population‐specific principal component analyses (PCAs) of multilocus genetic variation in *Polyommatus icarus*. Individuals are distinguished by sex. No apparent separation between male and female individuals was observed across populations.
**Figure S2:** Population‐specific principal component analyses (PCAs) of multilocus genetic variation in *Conocephalus fuscus*. Individuals are distinguished by sex. No apparent separation between male and female individuals was observed across populations.
**Figure S3:** (A) Genome‐wide genetic diversity, measured as unbiased expected heterozygosity (*uH*_EXP_), and (B) pairwise population genetic differentiation, measured as *F*_ST_, for the three insect species studied. For *Polyommatus icarus* and *Melanargia galathea*, analyses shown here were conducted with sex‐linked sites retained. Boxes represent the interquartile range, with the horizontal line indicating the median. Different letters indicate significant differences between species.
**Figure S4:** Relationships between genome‐wide genetic diversity (*uH*_EXP_) and edge density (500 m radius) and arable cover for *Polyommatus icarus* (A, B) and *Melanargia galathea* (C, D), with sex‐linked sites retained. For *Polyommatus icarus* and *Melanargia galathea*, analyses shown here were conducted with sex‐linked sites retained. Solid lines represent predicted relationships from linear models and shaded areas indicate 95% confidence intervals. Points represent partial residuals. Significance levels: ns = *p* > 0.05; *p < 0.05.
**Figure S5:** Relationship between pairwise genetic distance, measured as *F*_ST_/(1 − *F*_ST_), and log‐transformed geographic distance for populations of (A) *Polyommatus icarus*, (B) *Melanargia galathea* and (C) *Conocephalus fuscus*. For *Polyommatus icarus* and *Melanargia galathea*, sex‐linked SNPs were excluded from the analyses; this was not possible for *Conocephalus fuscus* because a suitable reference genome was unavailable. Black lines represent fitted relationships and shaded areas indicate 95% confidence intervals. Significance levels: ns = *p* > 0.05; *p < 0.05.
**Figure S6:** Relationship between pairwise genetic distance, measured as *F*_ST_/(1 − *F*_ST_), and log‐transformed geographic distance for populations of (A) *Polyommatus icarus* and (B) *Melanargia galathea*, with sex‐linked SNPs retained. Black lines represent fitted relationships and shaded areas indicate 95% confidence intervals. Significance levels: ns = *p* > 0.05; *p < 0.05.
**Figure S7:** Genetic structure analyses based on discriminant analysis of principal components (DAPC) and cross‐entropy values across the range of *K* values tested with sNMF for (A) *Polyommatus icarus*, (B) *Melanargia galathea* and (C) *Conocephalus fuscus*. For *Polyommatus icarus* and *Melanargia galathea*, analyses shown here were conducted with sex‐linked SNPs retained. For *Conocephalus fuscus*, sex‐linked SNPs could not be identified and excluded because a suitable reference genome was unavailable. The *K* value with the lowest cross‐entropy score was taken as the best‐supported number of ancestral populations. In the DAPC plots, sampling populations are shown in different colours. Axes represent the first two discriminant functions, with labels indicating the proportion of variance explained. Ellipses indicate 95% confidence regions around population centroids.
**Figure S8:** Location of the Günz Valley within Germany (A), sampling sites (black circles), and effective migration surfaces for *Polyommatus icarus* (B, C), *Melanargia galathea* (D, E) and *Conocephalus fuscus* (F, G). For *Polyommatus icarus* and *Melanargia galathea*, analyses shown here were conducted with sex‐linked SNPs retained. For *Conocephalus fuscus*, sex‐linked SNPs could not be identified and excluded because a suitable reference genome was unavailable. Orange areas indicate lower effective migration than expected under an isolation‐by‐distance model (negative log migration rates, log(*m*)), whereas blue areas indicate higher effective migration than expected (positive log migration rates). The base map was obtained from OpenStreetMap; national borders of Germany were sourced from the deutschlandGeoJSON repository. OpenStreetMap data are licensed by the OpenStreetMap Foundation (OSMF) under the Open Database License (ODbL).
**Table S1:** eva70326‐sup‐0001‐Supinfo.docx. *Polyommatus icarus* population ID, geographic coordinates, sample size before and after individual filtering, number of males and females, genome‐wide unbiased expected heterozygosity (*uH*_EXP_) and landscape variables used in the genetic‐diversity analyses. *uH*_EXP_ is shown for the main dataset excluding sex‐linked sites and, for comparison, for the dataset retaining sex‐linked sites.
**Table S2:** eva70326‐sup‐0001‐Supinfo.docx. *Melanargia galathea* population ID, geographic coordinates, sample size before and after individual filtering, genome‐wide unbiased expected heterozygosity (*uH*_EXP_) and landscape variables used in the genetic‐diversity analyses. *uH*_EXP_ is shown for the main dataset excluding sex‐linked sites and, for comparison, for the dataset retaining sex‐linked sites. Individual sex was not recorded for this species.
**Table S3:** eva70326‐sup‐0001‐Supinfo.docx. *Conocephalus fuscus* population ID, geographic coordinates, sample size before and after individual filtering, number of males and females, genome‐wide unbiased expected heterozygosity (*uH*_EXP_) and landscape variables used in the genetic‐diversity analyses.
**Table S4:** eva70326‐sup‐0001‐Supinfo.docx. *Polyommatus icarus* sequencing summary for individuals retained after individual‐level missingness filtering, including number of reads, number of RAD loci and average sequencing depth per individual.
**Table S5:** eva70326‐sup‐0001‐Supinfo.docx. *Melanargia galathea* sequencing summary for individuals retained after individual‐level missingness filtering, including number of reads, number of RAD loci and average sequencing depth per individual.
**Table S6:** eva70326‐sup‐0001‐Supinfo.docx. *Conocephalus fuscus* sequencing summary for individuals retained after individual‐level missingness filtering, including number of reads, number of RAD loci and average sequencing depth per individual.
**Table S7:** Number of genomic sites retained during preparation of the analysis‐specific datasets for each species. The genome‐wide genetic diversity dataset contained both polymorphic and monomorphic sites, whereas the dataset used for genetic differentiation and population structure analyses contained only polymorphic SNPs. For *P. icarus* and *M. galathea*, the reported numbers correspond to datasets after exclusion of sex‐linked sites. Counts are reported for filtering stages for which retained‐site numbers were recorded.
**Table S8:** Correlation coefficients between landscape predictors and genome‐wide genetic diversity (*uH*_EXP_) for the three focal species. Highlighted values indicate the spatial radius with the largest absolute correlation coefficient for each predictor and species.
**Table S9:** eva70326‐sup‐0001‐Supinfo.docx. *Polyommatus icarus* estimated pairwise *F*_ST_ between all location pairs (below diagonal). *p*‐values from 1000 bootstrap replicates (above diagonal).
**Table S10:** eva70326‐sup‐0001‐Supinfo.docx. *Melanargia galathea* estimated pairwise *F*_ST_ between all location pairs (below diagonal). *p*‐values from 1000 bootstrap replicates (above diagonal).
**Table S11:** eva70326‐sup‐0001‐Supinfo.docx. *Conocephalus fuscus* estimated pairwise *F*_ST_ between all location pairs (below diagonal). *p*‐values from 1000 bootstrap replicates (above diagonal).

## Data Availability

The raw genome sequencing data generated in this study have been deposited in the NCBI Sequence Read Archive (SRA) under BioProject accessions PRJNA1271996, PRJNA1272098 and PRJNA1271283. SNP data (VCF format) and scripts are available on figshare (https://doi.org/10.6084/m9.figshare.30784838). Command line scripts are also available on github (https://github.com/panastheod/insect‐conservation‐grassland‐genomics).

## References

[eva70326-bib-0001] Aleksanov, V. 2025. “Orthoptera Colonising the Urban Landscape and Urban Lawns: A Case of a Middle‐Russian City.” Ecology and Diversity 2, no. 2: 10004. 10.70322/ecoldivers.2025.10004.

[eva70326-bib-0002] Allendorf, F. W. , W. C. Funk , S. N. Aitken , M. Byrne , G. Luikart , and A. Antunes . 2022. Conservation and the Genomics of Populations. 3rd ed. University PressOxford. 10.1093/oso/9780198856566.001.0001.

[eva70326-bib-0003] Andersen, L. W. , K. Fog , and C. Damgaard . 2004. “Habitat Fragmentation Causes Bottlenecks and Inbreeding in the European Tree Frog ( *Hyla arborea* ).” Proceedings of the Royal Society of London. Series B: Biological Sciences 271, no. 1545: 1293–1302. 10.1098/rspb.2004.2720. PMC1691722 15306354

[eva70326-bib-0004] Arancibia, F. , R. C. Motta , and P. Clausing . 2020. “The Neglected Burden of Agricultural Intensification: A Contribution to the Debate on Land‐Use Change.” Journal of Land Use Science 15, no. 2–3: 235–251. 10.1080/1747423X.2019.1659431.

[eva70326-bib-0005] Avci, C. , and M. S. Taylan . 2023. “Bioacoustics of Tettigoniidae (Insecta, Orthoptera) Distributed in Hakkari (Eastern Anatolia) Province.” Journal of the Entomological Research Society 25: 371–386. 10.51963/jers.2023.95.

[eva70326-bib-0006] Balkenhol, N. 2015. Landscape Genetics: Concepts, Methods, Applications. Wiley Blackwell.

[eva70326-bib-0007] Baltz, L. M. , H. Gardein , H. Greil , R. J. Paxton , and P. Theodorou . 2026. “Different Paths, Similar Pressures: Divergent Drivers of Genetic Diversity Despite Convergent Genomic Signatures of Selection in Response to Urban Intensity in Two Oligolectic Bee Species.” Molecular Ecology 35, no. 9: e70370. 10.1111/mec.70370. PMC13156532 42104688

[eva70326-bib-0008] Bartoń, K. 2010. “MuMIn: Multi‐Model Inference.” p. 1.48.11 [Dataset]. 10.32614/CRAN.package.MuMIn.

[eva70326-bib-0011] Bellmann, H. , F. Rutschmann , C. Roesti , and A. Hochkirch . 2019. Der Kosmos Heuschreckenführer (Neuauflage). Kosmos.

[eva70326-bib-0012] Benton, T. G. , J. A. Vickery , and J. D. Wilson . 2003. “Farmland Biodiversity: Is Habitat Heterogeneity the Key?” Trends in Ecology & Evolution 18, no. 4: 182–188. 10.1016/S0169-5347(03)00011-9.

[eva70326-bib-0013] Brebner, J. S. , J. C. Makinson , O. K. Bates , et al. 2021. “Bumble Bees Strategically Use Ground Level Linear Features in Navigation.” Animal Behaviour 179: 147–160. 10.1016/j.anbehav.2021.07.003.

[eva70326-bib-0014] Brower, L. P. , O. R. Taylor , E. H. Williams , D. A. Slayback , R. R. Zubieta , and M. I. Ramírez . 2012. “Decline of Monarch Butterflies Overwintering in Mexico: Is the Migratory Phenomenon at Risk?” Insect Conservation and Diversity 5, no. 2: 95–100. 10.1111/j.1752-4598.2011.00142.x.

[eva70326-bib-0015] Brown, M. J. F. , and R. J. Paxton . 2009. “The Conservation of Bees: A Global Perspective.” Apidologie 40, no. 3: 410–416. 10.1051/apido/2009019.

[eva70326-bib-0016] Bushnell, B. 2014. “BBMap: A Fast, Accurate, Splice‐Aware Aligner.” Lawrence Berkeley National Laboratory. LBNL Report, LBNL‐7065E, 3.

[eva70326-bib-0017] Cardoso, P. , P. S. Barton , K. Birkhofer , et al. 2020. “Scientists' Warning to Humanity on Insect Extinctions.” Biological Conservation 242: 108426. 10.1016/j.biocon.2020.108426.

[eva70326-bib-0018] Cardoso, P. , T. L. Erwin , P. A. V. Borges , and T. R. New . 2011. “The Seven Impediments in Invertebrate Conservation and How to Overcome Them.” Biological Conservation 144, no. 11: 2647–2655. 10.1016/j.biocon.2011.07.024.

[eva70326-bib-0019] Chisté, M. N. , K. Mody , M. M. Gossner , et al. 2016. “Losers, Winners, and Opportunists: How Grassland Land‐Use Intensity Affects Orthopteran Communities.” Ecosphere 7, no. 11: e01545. 10.1002/ecs2.1545.

[eva70326-bib-0020] Chowdhury, S. , M. D. Jennions , M. P. Zalucki , M. Maron , J. E. M. Watson , and R. A. Fuller . 2023. “Protected Areas and the Future of Insect Conservation.” Trends in Ecology & Evolution 38, no. 1: 85–95. 10.1016/j.tree.2022.09.004. 36208964

[eva70326-bib-0021] Chowdhury, S. , M. P. Zalucki , J. O. Hanson , et al. 2023. “Three‐Quarters of Insect Species Are Insufficiently Represented by Protected Areas.” One Earth 6, no. 2: 139–146. 10.1016/j.oneear.2022.12.003.

[eva70326-bib-0022] Clarke, S. H. , E. R. Lawrence , J. Matte , et al. 2024. “Global Assessment of Effective Population Sizes: Consistent Taxonomic Differences in Meeting the 50/500 Rule.” Molecular Ecology 33, no. 11: e17353. 10.1111/mec.17353. 38613250

[eva70326-bib-0023] Coates, D. J. , M. Byrne , and C. Moritz . 2018. “Genetic Diversity and Conservation Units: Dealing With the Species‐Population Continuum in the Age of Genomics.” Frontiers in Ecology and Evolution 6: 165. 10.3389/fevo.2018.00165.

[eva70326-bib-0024] Cowley, M. J. R. , C. D. Thomas , D. B. Roy , et al. 2001. “Density–Distribution Relationships in British Butterflies. I. The Effect of Mobility and Spatial Scale.” Journal of Animal Ecology 70, no. 3: 410–425. 10.1046/j.1365-2656.2001.00508.x.

[eva70326-bib-0025] Crossley, M. S. , C. E. Latimer , C. M. Kennedy , and W. E. Snyder . 2023. “Past and Recent Farming Degrades Aquatic Insect Genetic Diversity.” Molecular Ecology 32, no. 13: 3356–3367. 10.1111/mec.16590. 35771845

[eva70326-bib-0026] Danecek, P. , A. Auton , G. Abecasis , et al. 2011. “The Variant Call Format and VCFtools.” Bioinformatics 27, no. 15: 2156–2158. 10.1093/bioinformatics/btr330. PMC3137218 21653522

[eva70326-bib-0027] Della Rocca, F. , E. Repetto , L. De Caria , and P. Milanesi . 2025. “Identifying Ecological Corridors of the Bush Cricket *Saga pedo* in Fragmented Landscapes.” Insects 16, no. 3: 279. 10.3390/insects16030279. PMC11943009 40266765

[eva70326-bib-0028] Detzel, P. (With Landesanstalt für Umweltschutz Baden‐Württemberg). 1998. Die Heuschrecken Baden‐Württembergs: Im Rahmen des Artenschutzprogrammes Baden‐Württemberg. Eugen Ulmer GmbH & Co.

[eva70326-bib-0029] Dray, S. , and A.‐B. Dufour . 2007. “The ade4 Package: Implementing the Duality Diagram for Ecologists.” Journal of Statistical Software 22, no. 4: 1–20. 10.18637/jss.v022.i04.

[eva70326-bib-0030] Eaton, D. A. R. , and I. Overcast . 2020. “ipyrad: Interactive Assembly and Analysis of RADseq Datasets.” Bioinformatics 36, no. 8: 2592–2594. 10.1093/bioinformatics/btz966. 31904816

[eva70326-bib-0031] Ellegren, H. , and N. Galtier . 2016. “Determinants of Genetic Diversity.” Nature Reviews Genetics 17, no. 7: 422–433. 10.1038/nrg.2016.58. 27265362

[eva70326-bib-0032] Ellis, E. C. , N. Gauthier , K. Klein Goldewijk , et al. 2021. “People Have Shaped Most of Terrestrial Nature for at Least 12,000 Years.” Proceedings of the National Academy of Sciences of the United States of America 118, no. 17: e2023483118. 10.1073/pnas.2023483118. PMC8092386 33875599

[eva70326-bib-0033] Emmet, A. M. , and J. Heath , eds. 1989. Hesperiidae—Nymphalidae: The Butterflies of Britain and Ireland. Harley.

[eva70326-bib-0034] Evans, D. 2012. “Building the European Union's Natura 2000 Network.” Nature Conservation 1: 11–26. 10.3897/natureconservation.1.1808.

[eva70326-bib-0035] Fahrig, L. 2017. “Ecological Responses to Habitat Fragmentation Per Se.” Annual Review of Ecology, Evolution, and Systematics 48, no. 1: 1–23. 10.1146/annurev-ecolsys-110316-022612.

[eva70326-bib-0036] Fartmann, T. , E. Jedicke , M. Streitberger , and G. Stuhldreher . 2021. Insektensterben in Mitteleuropa: Ursachen und Gegenmaßnahmen. Verlag Eugen Ulmer.

[eva70326-bib-0037] Frankham, R. , J. D. Ballou , D. A. Briscoe , and K. H. McInnes . 2002. Introduction to Conservation Genetics. Cambridge University Press.

[eva70326-bib-0038] Frichot, E. , and O. François . 2015. “LEA: An R Package for Landscape and Ecological Association Studies.” Methods in Ecology and Evolution 6, no. 8: 925–929. 10.1111/2041-210X.12382.

[eva70326-bib-0039] Fumy, F. , and T. Fartmann . 2023. “Low‐Intensity Land Use Fosters Species Richness of Threatened Butterflies and Grasshoppers in Mires and Grasslands.” Global Ecology and Conservation 41: e02357. 10.1016/j.gecco.2022.e02357.

[eva70326-bib-0040] Fumy, F. , C. Schwarz , and T. Fartmann . 2023. “Intensity of Grassland Management and Landscape Heterogeneity Determine Species Richness of Insects in Fragmented Hay Meadows.” Global Ecology and Conservation 47: e02672. 10.1016/j.gecco.2023.e02672.

[eva70326-bib-0041] Gardiner, T. 2010. “Precipitation and Habitat Degradation Influence the Occurrence of the Common Green Grasshopper *Omocestus viridulus* in Southeastern England.” Journal of Orthoptera Research 19, no. 2: 315–326. 10.1665/034.019.0219.

[eva70326-bib-0042] Gaublomme, E. , K. Maebe , K. Van Doninck , et al. 2013. “Loss of Genetic Diversity and Increased Genetic Structuring in Response to Forest Area Reduction in a Ground Dwelling Insect: A Case Study of the Flightless Carabid Beetle * carabus problematicus* (coleoptera, carabidae).” Insect Conservation and Diversity 6, no. 4: 473–482. 10.1111/icad.12002.

[eva70326-bib-0043] Gittleman, J. L. , and M. Kot . 1990. “Adaptation: Statistics and a Null Model for Estimating Phylogenetic Effects.” Systematic Zoology 39, no. 3: 227–241.

[eva70326-bib-0044] González‐Castellano, I. , N. Marí‐Mena , G. Segelbacher , et al. 2023. “Landscape Genetics of the Protected Spanish Moon Moth in Core, Buffer, and Peripheral Areas of the Ordesa y Monte Perdido National Park (Central Pyrenees, Spain).” Conservation Genetics 24, no. 6: 767–782. 10.1007/s10592-023-01536-z.

[eva70326-bib-0045] Goulson, D. 2019. “The Insect Apocalypse, and Why It Matters.” Current Biology 29, no. 19: R967–R971. 10.1016/j.cub.2019.06.069. 31593678

[eva70326-bib-0046] Gruber, B. , P. J. Unmack , O. F. Berry , and A. Georges . 2018. “Dartr: An r Package to Facilitate Analysis of SNP Data Generated From Reduced Representation Genome Sequencing.” Molecular Ecology Resources 18, no. 3: 691–699. 10.1111/1755-0998.12745. 29266847

[eva70326-bib-0047] Guariento, E. , J. Rüdisser , K. Fiedler , et al. 2023. “From Diverse to Simple: Butterfly Communities Erode From Extensive Grasslands to Intensively Used Farmland and Urban Areas.” Biodiversity and Conservation 32, no. 3: 867–882. 10.1007/s10531-022-02498-3.

[eva70326-bib-0048] Gutiérrez, D. , J. L. Le , R. Meniendez , R. J. Wilson , M. J. R. Cowley , and C. D. Thomas . 2001. “Metapopulations of Four Lepidopteran Herbivores on a Single Host Plant, *Lotus corniculatus*.”

[eva70326-bib-0049] Habel, J. C. , J. O. Engler , D. Rödder , and T. Schmitt . 2012. “Landscape Genetics of a Recent Population Extirpation in a Burnet Moth Species.” Conservation Genetics 13, no. 1: 247–255. 10.1007/s10592-011-0280-3.

[eva70326-bib-0050] Habel, J. C. , M. Junker , and T. Schmitt . 2010. “High Dispersal Ability and Low Genetic Differentiation in the Widespread Butterfly Species *Melanargia galathea* .” Journal of Insect Conservation 14, no. 5: 467–478. 10.1007/s10841-010-9275-5.

[eva70326-bib-0051] Habel, J. C. , M. J. Samways , and T. Schmitt . 2019. “Mitigating the Precipitous Decline of Terrestrial European Insects: Requirements for a New Strategy.” Biodiversity and Conservation 28, no. 6: 1343–1360. 10.1007/s10531-019-01741-8.

[eva70326-bib-0052] Hallmann, C. A. , M. Sorg , E. Jongejans , et al. 2017. “More Than 75 Percent Decline Over 27 Years in Total Flying Insect Biomass in Protected Areas.” PLoS One 12, no. 10: e0185809. 10.1371/journal.pone.0185809. PMC5646769 29045418

[eva70326-bib-0053] Hannappel, I. , and K. Fischer . 2020. “Grassland Intensification Strongly Reduces Butterfly Diversity in the Westerwald Mountain Range, Germany.” Journal of Insect Conservation 24, no. 2: 279–285. 10.1007/s10841-019-00195-1.

[eva70326-bib-0054] Hartig, F. 2024. “Package ‘DHARMa’: Residual Diagnostics for Hierarchical (Multi‐Level/Mixed) Regression Models (Version Version 0.4.7)” [Computer Software]. https://CRAN.R‐project.org/package=DHARMa.

[eva70326-bib-0055] Hemberger, J. , O. M. Bernauer , H. R. Gaines‐Day , and C. Gratton . 2023. “Landscape‐Scale Floral Resource Discontinuity Decreases Bumble Bee Occurrence and Alters Community Composition.” Ecological Applications 33, no. 7: e2907. 10.1002/eap.2907. 37602909

[eva70326-bib-0056] Hill, J. K. , C. L. Hughes , C. Dytham , and J. B. Searle . 2006. “Genetic Diversity in Butterflies: Interactive Effects of Habitat Fragmentation and Climate‐Driven Range Expansion.” Biology Letters 2, no. 1: 152–154. 10.1098/rsbl.2005.0401. PMC1617171 17148351

[eva70326-bib-0057] Höglund, J. 2009. Evolutionary Conservation Genetics. Oxford University Press.

[eva70326-bib-0058] Holderegger, R. , and H. H. Wagner . 2008. “Landscape Genetics.” Bioscience 58, no. 3: 199–207. 10.1641/B580306.

[eva70326-bib-0059] Hopfenmüller, S. , P. Guggenberger‐Waibel , and G. Weber . 2015. “DBU‐Projekt ‘Naturschutz in Intensivgrünland‐Regionen—Biotopverbund am Beispiel des Günztals’.” ANLiegen Natur 37, no. 2: 97–100.

[eva70326-bib-0060] Jacobson, A. P. , J. Riggio , A. M. Tait , and J. E. M. Baillie . 2019. “Global Areas of Low Human Impact (‘Low Impact Areas’) and Fragmentation of the Natural World.” Scientific Reports 9, no. 1: 14179. 10.1038/s41598-019-50558-6. PMC6775135 31578431

[eva70326-bib-0061] James, G. , D. Witten , T. Hastie , and R. Tibshirani . 2013. An Introduction to Statistical Learning: With Applications in R. Vol. 103. Springer New York. 10.1007/978-1-4614-7138-7.

[eva70326-bib-0062] Jauker, F. , T. Diekötter , F. Schwarzbach , and V. Wolters . 2009. “Pollinator Dispersal in an Agricultural Matrix: Opposing Responses of Wild Bees and Hoverflies to Landscape Structure and Distance From Main Habitat.” Landscape Ecology 24, no. 4: 547–555. 10.1007/s10980-009-9331-2.

[eva70326-bib-0063] Jerrentrup, J. S. , N. Wrage‐Mönnig , K. Röver , and J. Isselstein . 2014. “Grazing Intensity Affects Insect Diversity via Sward Structure and Heterogeneity in a Long‐Term Experiment.” Journal of Applied Ecology 51, no. 4: 968–977. 10.1111/1365-2664.12244.

[eva70326-bib-0064] Jha, S. 2015. “Contemporary Human‐Altered Landscapes and Oceanic Barriers Reduce Bumble Bee Gene Flow.” Molecular Ecology 24, no. 5: 993–1006. 10.1111/mec.13090. 25626470

[eva70326-bib-0065] Jombart, T. 2008. “Adegenet: A R Package for the Multivariate Analysis of Genetic Markers.” Bioinformatics 24, no. 11: 1403–1405. 10.1093/bioinformatics/btn129. 18397895

[eva70326-bib-0066] Jombart, T. , and I. Ahmed . 2011. “Adegenet 1.3‐1: New Tools for the Analysis of Genome‐Wide SNP Data.” Bioinformatics 27, no. 21: 3070–3071. 10.1093/bioinformatics/btr521. PMC3198581 21926124

[eva70326-bib-0067] Landis, D. A. 2017. “Designing Agricultural Landscapes for Biodiversity‐Based Ecosystem Services.” Basic and Applied Ecology 18: 1–12. 10.1016/j.baae.2016.07.005.

[eva70326-bib-0068] Langhammer, P. F. , J. W. Bull , J. E. Bicknell , et al. 2024. “The Positive Impact of Conservation Action.” Science 384, no. 6694: 453–458. 10.1126/science.adj6598. 38662833

[eva70326-bib-0069] Lenth, R. V. , and J. Piaskowski . 2025. “emmeans: Estimated Marginal Means, aka Least‐Squares Means.” p. 2.0.0 [Dataset]. 10.32614/CRAN.package.emmeans.

[eva70326-bib-0070] Löffler, F. , D. Poniatowski , and T. Fartmann . 2019. “Orthoptera Community Shifts in Response to Land‐Use and Climate Change—Lessons From a Long‐Term Study Across Different Grassland Habitats.” Biological Conservation 236: 315–323. 10.1016/j.biocon.2019.05.058.

[eva70326-bib-0071] López‐Uribe, M. M. , S. Jha , and A. Soro . 2019. “A Trait‐Based Approach to Predict Population Genetic Structure in Bees.” Molecular Ecology 28, no. 8: 1919–1929. 10.1111/mec.15028. 30667117

[eva70326-bib-0072] Mallinoff, J. A. , R. Petric , C. A. Diggins , et al. 2025. “Ecotone Might Provide Key Refugium for Sky Island Mammals in the Southern Appalachian Mountains.” Ecology and Evolution 15, no. 10: e72374. 10.1002/ece3.72374. PMC12568775 41169306

[eva70326-bib-0073] Manel, S. , M. K. Schwartz , G. Luikart , and P. Taberlet . 2003. “Landscape Genetics: Combining Landscape Ecology and Population Genetics.” Trends in Ecology & Evolution 18, no. 4: 189–197. 10.1016/S0169-5347(03)00008-9.

[eva70326-bib-0074] Manichaikul, A. , J. C. Mychaleckyj , S. S. Rich , K. Daly , M. Sale , and W.‐M. Chen . 2010. “Robust Relationship Inference in Genome‐Wide Association Studies.” Bioinformatics 26, no. 22: 2867–2873. 10.1093/bioinformatics/btq559. PMC3025716 20926424

[eva70326-bib-0075] Martin, E. A. , M. Dainese , Y. Clough , et al. 2019. “The Interplay of Landscape Composition and Configuration: New Pathways to Manage Functional Biodiversity and Agroecosystem Services Across Europe.” Ecology Letters 22, no. 7: 1083–1094. 10.1111/ele.13265. 30957401

[eva70326-bib-0076] Martinez, J. G. , J. Carranza , J. L. Fernandez‐Garcia , and C. B. Sanchez‐Prieto . 2002. “Genetic Variation of Red Deer Populations Under Hunting Exploitation in Southwestern Spain.” Journal of Wildlife Management 66, no. 4: 1273. 10.2307/3802960.

[eva70326-bib-0077] Morán‐López, R. , E. Cortés Gañán , O. Uceda Tolosa , and J. M. Sánchez Guzmán . 2020. “The Umbrella Effect of Natura 2000 Annex Species Spreads Over Multiple Taxonomic Groups, Conservation Attributes and Organizational Levels.” Animal Conservation 23, no. 4: 407–419. 10.1111/acv.12551.

[eva70326-bib-0078] Nolen, Z. J. , P. Jamelska , A. S. T. Lara , N. Wahlberg , and A. Runemark . 2026. “Species‐Specific Loss of Genetic Diversity and Inbreeding Following Agricultural Intensification.” Genome Biology and Evolution: evag235. 10.1093/gbe/evag235.42781869

[eva70326-bib-0079] Oksanen, J. , G. L. Simpson , F. G. Blanchet , et al. 2026. “vegan: Community Ecology Package.” p. 2.7‐5. [Dataset]. 10.32614/CRAN.package.vegan.

[eva70326-bib-0080] Packer, L. , A. Zayed , J. C. Grixti , et al. 2005. “Conservation Genetics of Potentially Endangered Mutualisms: Reduced Levels of Genetic Variation in Specialist Versus Generalist Bees.” Conservation Biology 19, no. 1: 195–202. 10.1111/j.1523-1739.2005.00601.x.

[eva70326-bib-0081] Pembleton, L. W. , N. O. I. Cogan , and J. W. Forster . 2013. “StAMPP: An R Package for Calculation of Genetic Differentiation and Structure of Mixed‐Ploidy Level Populations.” Molecular Ecology Resources 13, no. 5: 946–952. 10.1111/1755-0998.12129. 23738873

[eva70326-bib-0082] Pérez‐Ruzafa, Á. , M. González‐Wangüemert , P. Lenfant , C. Marcos , and J. A. García‐Charton . 2006. “Effects of Fishing Protection on the Genetic Structure of Fish Populations.” Biological Conservation 129, no. 2: 244–255. 10.1016/j.biocon.2005.10.040.

[eva70326-bib-0083] Petkova, D. 2025. “reemsplots2: Generate Plots to Inspect and Visualize the Results of EEMS” [Computer Software]. https://github.com/dipetkov/reemsplots2.

[eva70326-bib-0084] Petkova, D. , J. Novembre , and M. Stephens . 2016. “Visualizing Spatial Population Structure With Estimated Effective Migration Surfaces.” Nature Genetics 48, no. 1: 94–100. 10.1038/ng.3464. PMC4696895 26642242

[eva70326-bib-0085] Purcell, S. , B. Neale , K. Todd‐Brown , et al. 2007. “PLINK: A Tool Set for Whole‐Genome Association and Population‐Based Linkage Analyses.” American Journal of Human Genetics 81, no. 3: 559–575. 10.1086/519795. PMC1950838 17701901

[eva70326-bib-0118] R Core Team . 2024. “R: A Language and Environment for Statistical Computing.” R Foundation for Statistical Computing, Vienna, Austria. https://www.R‐project.org/.

[eva70326-bib-0086] Raven, P. H. , and D. L. Wagner . 2021. “Agricultural Intensification and Climate Change Are Rapidly Decreasing Insect Biodiversity.” Proceedings of the National Academy of Sciences of the United States of America 118, no. 2: e2002548117. 10.1073/pnas.2002548117. PMC7812793 33431564

[eva70326-bib-0087] Reinhardt, K. , G. Köhler , S. Maas , and P. Detzel . 2005. “Low Dispersal Ability and Habitat Specificity Promote Extinctions in Rare but Not in Widespread Species: The Orthoptera of Germany.” Ecography 28, no. 5: 593–602. 10.1111/j.2005.0906-7590.04285.x.

[eva70326-bib-0088] Rivest, S. A. , and H. M. Kharouba . 2021. “Anthropogenic Disturbance Promotes the Abundance of a Newly Introduced Butterfly, the European Common Blue (*Polyommatus icarus*; Lepidoptera: Lycaenidae), in Canada.” Canadian Journal of Zoology 99, no. 8: 642–652. 10.1139/cjz-2021-0009.

[eva70326-bib-0089] Romiguier, J. , P. Gayral , M. Ballenghien , et al. 2014. “Comparative Population Genomics in Animals Uncovers the Determinants of Genetic Diversity.” Nature 515, no. 7526: 261–263. 10.1038/nature13685. 25141177

[eva70326-bib-0090] Samways, M. J. , P. S. Barton , K. Birkhofer , et al. 2020. “Solutions for Humanity on How to Conserve Insects.” Biological Conservation 242: 108427. 10.1016/j.biocon.2020.108427.

[eva70326-bib-0091] Schleimer, A. , and A. C. Frantz . 2025. “Landscape Influence on Pollinator Population Genetic Connectivity.” Insect Conservation and Diversity 18, no. 3: 285–302. 10.1111/icad.12813.

[eva70326-bib-0092] Schmidt, T. L. , M. Jasper , A. R. Weeks , and A. A. Hoffmann . 2021. “Unbiased Population Heterozygosity Estimates From Genome‐Wide Sequence Data.” Methods in Ecology and Evolution 12, no. 10: 1888–1898. 10.1111/2041-210X.13659.

[eva70326-bib-0093] Schmitt, T. , A. Giessl , and A. Seitz . 2003. “Did *Polyommatus icarus* (Lepidoptera: Lycaenidae) Have Distinct Glacial Refugia in Southern Europe? Evidence From Population Genetics: Biogeography of *P. icarus* .” Biological Journal of the Linnean Society 80, no. 3: 529–538. 10.1046/j.1095-8312.2003.00261.x.

[eva70326-bib-0094] Schwarz, C. , F. Fumy , M. Drung , and T. Fartmann . 2023. “Insect‐Friendly Harvest in Hay Meadows—Uncut Refuges Are of Vital Importance for Conservation Management.” Global Ecology and Conservation 48: e02731. 10.1016/j.gecco.2023.e02731.

[eva70326-bib-0095] Seibold, S. , M. M. Gossner , N. K. Simons , et al. 2019. “Arthropod Decline in Grasslands and Forests Is Associated With Landscape‐Level Drivers.” Nature 574, no. 7780: 671–674. 10.1038/s41586-019-1684-3. 31666721

[eva70326-bib-0096] Sharma, I. , and S. Birman . 2024. “Biodiversity Loss, Ecosystem Services, and Their Role in Promoting Sustainable Health.” In The Climate‐Health‐Sustainability Nexus: Understanding the Interconnected Impact on Populations and the Environment, edited by P. Singh and N. Yadav , 163–188. Springer Nature Switzerland. 10.1007/978-3-031-56564-9_7.

[eva70326-bib-0097] Shi, X. , C. Ma , J. De Kraker , et al. 2024. “Influence of Agricultural Intensification on Pollinator Pesticide Exposure, Food Acquisition and Diversity.” Journal of Applied Ecology 61, no. 8: 1905–1917. 10.1111/1365-2664.14701.

[eva70326-bib-0098] Sopniewski, J. , and R. A. Catullo . 2024. “Estimates of Heterozygosity From Single Nucleotide Polymorphism Markers Are Context‐Dependent and Often Wrong.” Molecular Ecology Resources 24, no. 4: e13947. 10.1111/1755-0998.13947. 38433491

[eva70326-bib-0099] Starnes, T. , A. E. Beresford , G. M. Buchanan , M. Lewis , A. Hughes , and R. D. Gregory . 2021. “The Extent and Effectiveness of Protected Areas in the UK.” Global Ecology and Conservation 30: e01745. 10.1016/j.gecco.2021.e01745.

[eva70326-bib-0100] Streiff, R. , P. Audiot , A. Foucart , M. Lecoq , and J.‐Y. Rasplus . 2006. “Genetic Survey of Two Endangered Grasshopper Subspecies, *Prionotropis hystrix rhodanica* and *Prionotropis hystrix azami* (Orthoptera, Pamphagidae): Within‐ and Between‐Population Dynamics at the Regional Scale.” Conservation Genetics 7: 331–344.

[eva70326-bib-0101] Sucháčková Bartoňová, A. , D. Linke , I. Klečková , P. G. Ribeiro , and P. Matos‐Maraví . 2023. “Incorporating Genomics Into Insect Conservation: Butterflies as a Model Group.” Insect Conservation and Diversity 16, no. 4: 427–440. 10.1111/icad.12643.

[eva70326-bib-0102] Suni, S. , and M. Hernandez . 2023. “Strong Decreases in Genetic Diversity Despite High Gene Flow for a Solitary Bee.” Conservation Genetics 24, no. 5: 607–615. 10.1007/s10592-023-01524-3.

[eva70326-bib-0103] Terzer, E. , M. Schmid , B. Bauert , et al. 2024. “Distinct Spatial Patterns of Genetic Structure and Diversity in the Butterfly Marbled White (*Melanargia galathea*) Inhabiting Fragmented Grasslands.” Conservation Genetics 25, no. 3: 631–645. 10.1007/s10592-023-01593-4.

[eva70326-bib-0104] Theodorou, P. , S.‐C. Herbst , B. Kahnt , et al. 2020. “Urban Fragmentation Leads to Lower Floral Diversity, With Knock‐On Impacts on Bee Biodiversity.” Scientific Reports 10, no. 1: 21756. 10.1038/s41598-020-78736-x. PMC7730174 33303909

[eva70326-bib-0105] Theodorou, P. , O. Kühn , L. M. Baltz , et al. 2022. “Bumble Bee Colony Health and Performance Vary Widely Across the Urban Ecosystem.” Journal of Animal Ecology 91, no. 10: 2135–2148. 10.1111/1365-2656.13797. 36002939

[eva70326-bib-0106] Theodorou, P. , R. Radzevičiūtė , G. Lentendu , et al. 2020. “Urban Areas as Hotspots for Bees and Pollination but Not a Panacea for All Insects.” Nature Communications 11, no. 1: 576. 10.1038/s41467-020-14496-6. PMC6989530 31996690

[eva70326-bib-0107] Thompson, C. E. P. , T. A. Pelletier , and B. C. Carstens . 2021. “Genetic Diversity of North American Vertebrates in Protected Areas.” Biological Journal of the Linnean Society 132, no. 2: 388–399. 10.1093/biolinnean/blaa195.

[eva70326-bib-0108] Tolman, T. , and R. Lewington , eds. 1997. Butterflies of Britain & Europe. HarperCollins.

[eva70326-bib-0109] Van Klink, R. , D. E. Bowler , K. B. Gongalsky , M. Shen , S. R. Swengel , and J. M. Chase . 2024. “Disproportionate Declines of Formerly Abundant Species Underlie Insect Loss.” Nature 628, no. 8007: 359–364. 10.1038/s41586-023-06861-4. PMC11006610 38123681

[eva70326-bib-0110] Van Klink, R. , D. E. Bowler , K. B. Gongalsky , A. B. Swengel , A. Gentile , and J. M. Chase . 2020. “Meta‐Analysis Reveals Declines in Terrestrial but Increases in Freshwater Insect Abundances.” Science 368, no. 6489: 417–420. 10.1126/science.aax9931. 32327596

[eva70326-bib-0111] Vanbergen, A. J. , and The Insect Pollinators Initiative . 2013. “Threats to an Ecosystem Service: Pressures on Pollinators.” Frontiers in Ecology and the Environment 11, no. 5: 251–259. 10.1890/120126.

[eva70326-bib-0112] Vandewoestijne, S. , T. Martin , S. Liégeois , and M. Baguette . 2004. “Dispersal, Landscape Occupancy and Population Structure in the Butterfly *Melanargia galathea* .” Basic and Applied Ecology 5, no. 6: 581–591. 10.1016/j.baae.2004.07.004.

[eva70326-bib-0119] Voith, J. , A. Beckmann , J. Sachteleben , H. Schlumprecht , and G. Waeber . 2016b. “Rote Liste und Gesamtartenliste der Heuschrecken (Saltatoria) Bayerns.” Bayerisches Landesamt für Umwelt (LfU).

[eva70326-bib-0120] Voith, J. , M. Bräu , M. Dolek , A. Nunner , and W. Wolf . 2016a. “Rote Liste und Gesamtartenliste der Tagfalter (Lepidoptera: Rhopalocera) Bayerns.” Bayerisches Landesamt für Umwelt (LfU).

[eva70326-bib-0113] Wagner, D. L. 2020. “Insect Declines in the Anthropocene.” Annual Review of Entomology 65: 457–480. 10.1146/annurev-ento-011019-025151 31610138

[eva70326-bib-0114] Wagner, D. L. , E. M. Grames , M. L. Forister , M. R. Berenbaum , and D. Stopak . 2021. “Insect Decline in the Anthropocene: Death by a Thousand Cuts.” Proceedings of the National Academy of Sciences of the United States of America 118, no. 2: e2023989118. 10.1073/pnas.2023989118. PMC7812858 33431573

[eva70326-bib-0115] Waples, R. S. 2025. “The Idiot's Guide to Effective Population Size.” Molecular Ecology 34, no. 23: e17670. 10.1111/mec.17670. 39925199

[eva70326-bib-0116] Webster, M. T. , A. Beaurepaire , P. Neumann , and E. Stolle . 2023. “Population Genomics for Insect Conservation.” Annual Review of Animal Biosciences 11, no. 1: 115–140. 10.1146/annurev-animal-122221-075025. 36375448

[eva70326-bib-0117] Weyer, J. , J. Weinberger , and A. Hochkirch . 2012. “Mobility and Microhabitat Utilization in a Flightless Wetland Grasshopper, *Chorthippus montanus* (Charpentier, 1825).” Journal of Insect Conservation 16, no. 3: 379–390. 10.1007/s10841-011-9423-6.

