# Supplementary material for: Edge‐Rich Landscapes Promote Genetic Diversity While Arable Land Restricts It: Landscape‐Genomic Patterns Across Three Insect Species in an Agriculture‐Dominated Grassland Mosaic
Source: Evol Appl. 2026 Oct 1;19(10):e70326. doi: 10.1111/eva.70326 (PMC13629290; doi:10.1111/eva.70326)
Supplement: Supplementary file 1 [file EVA-19-e70326-s001.docx]

**Supplementary Information**

**Figure S1.** Population-specific principal component analyses (PCAs) of multilocus genetic variation in Polyommatus icarus. Individuals are distinguished by sex. No apparent separation between male and female individuals was observed across populations.

**Figure S2.** Population-specific principal component analyses (PCAs) of multilocus genetic variation in *Conocephalus fuscus*. Individuals are distinguished by sex. No apparent separation between male and female individuals was observed across populations.

**Figure S3.** (A) Genome-wide genetic diversity, measured as unbiased expected heterozygosity (*uH*_EXP_), and (B) pairwise population genetic differentiation, measured as *F*_ST_, for the three insect species studied. For *Polyommatus icarus* and *Melanargia galathea*, analyses shown here were conducted with sex-linked sites retained. Boxes represent the interquartile range, with the horizontal line indicating the median. Different letters indicate significant differences between species.

**Figure S4.** Relationships between genome-wide genetic diversity (*uH*_EXP_) and edge density (500 m radius) and arable cover for *Polyommatus icarus* (A, B) and *Melanargia galathea* (C, D), with sex-linked sites retained. For *Polyommatus icarus* and *Melanargia galathea*, analyses shown here were conducted with sex-linked sites retained. Solid lines represent predicted relationships from linear models and shaded areas indicate 95% confidence intervals. Points represent partial residuals. Significance levels: ns = p > 0.05; * = p < 0.05.

**Figure S5.** Relationship between pairwise genetic distance, measured as FST/(1 - FST), and log-transformed geographic distance for populations of (A) *Polyommatus icarus*, (B) *Melanargia galathea* and (C) *Conocephalus fuscus*. For *Polyommatus icarus* and *Melanargia galathea*, sex-linked SNPs were excluded from the analyses; this was not possible for *Conocephalus fuscus* because a suitable reference genome was unavailable.  Black lines represent fitted relationships and shaded areas indicate 95% confidence intervals. Significance levels: ns = p > 0.05; * = p < 0.05.

**Figure S6.** Relationship between pairwise genetic distance, measured as FST/(1 - FST), and log-transformed geographic distance for populations of (A) *Polyommatus icarus* and (B) *Melanargia galathea*, with sex-linked SNPs retained. Black lines represent fitted relationships and shaded areas indicate 95% confidence intervals. Significance levels: ns = p > 0.05; * = p < 0.05.

**Figure S7.** Genetic structure analyses based on discriminant analysis of principal components (DAPC) and cross-entropy values across the range of K values tested with sNMF for (A) *Polyommatus icarus*, (B) *Melanargia galathea* and (C) *Conocephalus fuscus*. For *Polyommatus icarus* and *Melanargia galathea*, analyses shown here were conducted with sex-linked SNPs retained. For *Conocephalus fuscus*, sex-linked SNPs could not be identified and excluded because a suitable reference genome was unavailable. The K value with the lowest cross-entropy score was taken as the best-supported number of ancestral populations. In the DAPC plots, sampling populations are shown in different colours. Axes represent the first two discriminant functions, with labels indicating the proportion of variance explained. Ellipses indicate 95% confidence regions around population centroids.

**Figure S8.** Location of the Günz Valley within Germany (A), sampling sites (black circles), and effective migration surfaces for *Polyommatus icarus* (B, C), *Melanargia galathea* (D, E) and *Conocephalus fuscus* (F, G). For *Polyommatus icarus* and *Melanargia galathea*, analyses shown here were conducted with sex-linked SNPs retained. For *Conocephalus fuscus*, sex-linked SNPs could not be identified and excluded because a suitable reference genome was unavailable. Orange areas indicate lower effective migration than expected under an isolation-by-distance model (negative log migration rates, log(m)), whereas blue areas indicate higher effective migration than expected (positive log migration rates). The base map was obtained from OpenStreetMap; national borders of Germany were sourced from the deutschlandGeoJSON repository. OpenStreetMap data are licensed by the OpenStreetMap Foundation (OSMF) under the Open Database License (ODbL).

**Table S1.** *Polyommatus icarus* population ID, geographic coordinates, sample size before and after individual filtering, number of males and females, genome-wide unbiased expected heterozygosity (*uH*_EXP_), and landscape variables used in the genetic-diversity analyses. *uH*_EXP_ is shown for the main dataset excluding sex-linked sites and, for comparison, for the dataset retaining sex-linked sites.

| Site | Longitude | Latitude | N | N after filtering | *Males/Females* | *uH*_EXP_ | *uH*_EXP_ (incl. sex-chromosome SNPs) | Edge density (500 m radius) | Fragmentation of extensive meadows (700 m radius) | Proportion of arable cover (700 m radius) | Proportion of extensive meadows (1000 m radius) | Proportion of forest (1000 m radius) | Proportion of intensive meadows (500 m radius) |
| --- | --- | --- | --- | --- | --- | --- | --- | --- | --- | --- | --- | --- | --- |
| P1 | 10.31627956 | 48.32319648 | 9 | 6 | 5/1 | 0.00743 | 0.00743 | 0.0576 | 0.00055 | 0.30 | 0.25 | 0.12 | 0.06 |
| P2 | 10.30611626 | 48.35007293 | 16 | 12 | 8/4 | 0.00764 | 0.00765 | 0.0630 | 0.00018 | 0.16 | 0.04 | 0.09 | 0.46 |
| P3 | 10.33870475 | 48.07135725 | 10 | 9 | 6/3 | 0.00748 | 0.00747 | 0.0374 | 0.00028 | 0.19 | 0.15 | 0.33 | 0.22 |
| P4 | 10.32713676 | 48.03666464 | 10 | 6 | 5/1 | 0.00746 | 0.00745 | 0.0492 | 0.00037 | 0.14 | 0.04 | 0.15 | 0.45 |
| P5 | 10.28643787 | 47.98775896 | 8 | 8 | 7/1 | 0.00775 | 0.00775 | 0.0518 | 0.00044 | 0.00 | 0.24 | 0.54 | 0.01 |
| P7 | 10.36062431 | 47.8966619 | 10 | 10 | 4/6 | 0.00760 | 0.00761 | 0.0320 | 0.00024 | 0.03 | 0.23 | 0.09 | 0.51 |
| P8 | 10.36488976 | 47.93141031 | 14 | 14 | 8/6 | 0.00764 | 0.00764 | 0.0400 | 0.00017 | 0.07 | 0.39 | 0.15 | 0.40 |
| P9 | 10.41003513 | 47.88397311 | 13 | 13 | 8/5 | 0.00765 | 0.00765 | 0.0416 | 0.00036 | 0.00 | 0.32 | 0.50 | 0.00 |
| P10 | 10.39375154 | 47.90542134 | 10 | 9 | 6/3 | 0.00762 | 0.00763 | 0.0429 | 0.00026 | 0.02 | 0.33 | 0.34 | 0.16 |
| P11 | 10.40764789 | 47.86254979 | 10 | 10 | 8/2 | 0.00762 | 0.00763 | 0.0564 | 0.00032 | 0.00 | 0.48 | 0.35 | 0.04 |
| P12 | 10.4155423 | 47.82549973 | 11 | 10 | 6/4 | 0.00748 | 0.00748 | 0.0381 | 0.00013 | 0.01 | 0.46 | 0.09 | 0.30 |
| P13 | 10.27436758 | 48.21571908 | 15 | 14 | 12/2 | 0.00765 | 0.00765 | 0.0561 | 0.00026 | 0.20 | 0.26 | 0.08 | 0.16 |
| P15 | 10.24215294 | 48.18952053 | 9 | 8 | 8/0 | 0.00748 | 0.00749 | 0.0273 | 0.00039 | 0.03 | 0.14 | 0.66 | 0.01 |
| P16 | 10.33714281 | 48.17284595 | 10 | 9 | 3/6 | 0.00756 | 0.00756 | 0.0316 | 0.00022 | 0.07 | 0.02 | 0.60 | 0.31 |

**Table S2.** *Melanargia galathea* population ID, geographic coordinates, sample size before and after individual filtering, genome-wide unbiased expected heterozygosity (*uH*_EXP_), and landscape variables used in the genetic-diversity analyses. *uH*_EXP_ is shown for the main dataset excluding sex-linked sites and, for comparison, for the dataset retaining sex-linked sites. Individual sex was not recorded for this species.

| Site | Longitude | Latitude | N | N after filtering | *Males/Females* | *uH*_EXP_ | *uH*_EXP_ (incl. sex-chromosome SNPs) | Edge density (500 m radius) | Fragmentation of extensive meadows (1000 m radius) | Proportion of arable cover (700 m radius) | Proportion of extensive meadows (1000 m radius) | Proportion of intensive meadows (1000 m radius) | Proportion of forest (1000 m radius) |
| --- | --- | --- | --- | --- | --- | --- | --- | --- | --- | --- | --- | --- | --- |
| M7 | 10.36062431 | 47.8966619 | 10 | 10 | NA | 0.00643 | 0.006421 | 0.0320 | 0.00024 | 0.03 | 0.23 | 0.56 | 0.09 |
| M8 | 10.36488976 | 47.93141031 | 8 | 7 | NA | 0.006455 | 0.006444 | 0.0400 | 0.00013 | 0.07 | 0.39 | 0.36 | 0.15 |
| M9 | 10.41003513 | 47.88397311 | 11 | 11 | NA | 0.006591 | 0.006578 | 0.0416 | 0.00021 | 0.00 | 0.32 | 0.10 | 0.50 |
| M10 | 10.39375154 | 47.90542134 | 9 | 9 | NA | 0.006539 | 0.006528 | 0.0429 | 0.00020 | 0.02 | 0.33 | 0.21 | 0.34 |
| M11 | 10.40764789 | 47.86254979 | 9 | 9 | NA | 0.006665 | 0.006658 | 0.0564 | 0.00024 | 0.00 | 0.48 | 0.08 | 0.35 |
| M12 | 10.4155423 | 47.82549973 | 11 | 11 | NA | 0.006679 | 0.006672 | 0.0381 | 0.00012 | 0.01 | 0.46 | 0.37 | 0.09 |
| M15 | 10.24215294 | 48.18952053 | 10 | 10 | NA | 0.006336 | 0.006325 | 0.0273 | 0.00034 | 0.03 | 0.14 | 0.09 | 0.66 |
| M16 | 10.33714281 | 48.17284595 | 9 | 9 | NA | 0.006597 | 0.006587 | 0.0316 | 0.00031 | 0.07 | 0.02 | 0.24 | 0.60 |

**Table S3.** *Conocephalus fuscus* population ID, geographic coordinates, sample size before and after individual filtering, number of males and females, genome-wide unbiased expected heterozygosity (*uH*_EXP_), and landscape variables used in the genetic-diversity analyses.

| Site | Longitude | Latitude | N | N after filtering | *Males/Females* | *uH*_EXP_ | Edge density (500 m radius) | Fragmentation of extensive meadows (1000 m radius) | Proportion of arable (1000 m radius) | Proportion of extensive meadows (500 m radius) | Proportion of intensive meadows (700 m radius) | Proportion of forest (500 m radius) |
| --- | --- | --- | --- | --- | --- | --- | --- | --- | --- | --- | --- | --- |
| C1 | 10.31627956 | 48.32319648 | 13 | 8 | 5/3 | 0.00425 | 0.0576 | 0.00052 | 0.39 | 0.42 | 0.05 | 0.24 |
| C2 | 10.30806191 | 48.35055766 | 14 | 14 | 12/2 | 0.00436 | 0.0614 | 0.00023 | 0.31 | 0.08 | 0.34 | 0.12 |
| C3 | 10.33870475 | 48.07135725 | 3 | 3 | 2/1 | 0.00407 | 0.0374 | 0.00024 | 0.20 | 0.19 | 0.24 | 0.43 |
| C4 | 10.3236985 | 48.03611313 | 9 | 9 | 8/1 | 0.00426 | 0.0531 | 0.00015 | 0.11 | 0.02 | 0.39 | 0.08 |
| C5 | 10.28643787 | 47.98775896 | 12 | 12 | 8/4 | 0.00446 | 0.0518 | 0.00045 | 0.01 | 0.34 | 0.05 | 0.54 |
| C6 | 10.38121976 | 47.99144524 | 12 | 12 | 7/5 | 0.00408 | 0.0249 | 0.00030 | 0.06 | 0.03 | 0.64 | 0.25 |
| C7 | 10.36062431 | 47.8966619 | 10 | 10 | 5/5 | 0.00437 | 0.0320 | 0.00024 | 0.02 | 0.33 | 0.53 | 0.04 |
| C8 | 10.35767383 | 47.93338085 | 10 | 10 | 9/1 | 0.00410 | 0.0317 | 0.00014 | 0.04 | 0.30 | 0.50 | 0.07 |
| C9 | 10.41003513 | 47.88397311 | 11 | 11 | 9/2 | 0.00419 | 0.0416 | 0.00021 | 0.02 | 0.27 | 0.06 | 0.66 |
| C11 | 10.40764789 | 47.86254979 | 11 | 10 | 8/2 | 0.00425 | 0.0564 | 0.00024 | 0.00 | 0.55 | 0.06 | 0.30 |
| C12 | 10.4155423 | 47.82549973 | 11 | 11 | 7/4 | 0.00405 | 0.0381 | 0.00012 | 0.01 | 0.48 | 0.36 | 0.15 |
| C13 | 10.27436758 | 48.21571908 | 5 | 5 | 5/0 | 0.00429 | 0.0561 | 0.00026 | 0.22 | 0.31 | 0.18 | 0.07 |
| C14 | 10.3219575 | 48.20433599 | 10 | 10 | 10/0 | 0.00425 | 0.0437 | 0.00034 | 0.07 | 0.04 | 0.27 | 0.31 |
| C15 | 10.24215294 | 48.18952053 | 10 | 10 | 4/6 | 0.00402 | 0.0273 | 0.00034 | 0.07 | 0.19 | 0.03 | 0.72 |
| C16 | 10.33605047 | 48.17029246 | 12 | 12 | 8/4 | 0.00397 | 0.0326 | 0.00025 | 0.12 | 0.03 | 0.34 | 0.48 |

**Table S4.** *Polyommatus icarus* sequencing summary for individuals retained after individual-level missingness filtering, including number of reads, number of RAD loci and average sequencing depth per individual.

| Sample | Population | Reads (M) | Number of RAD loci | Average depth |
| --- | --- | --- | --- | --- |
| Pi1 | P1 | 2.50 | 61002 | 24.55 |
| Pi2 | P1 | 4.37 | 65634 | 39.28 |
| Pi4 | P1 | 4.05 | 61739 | 39.4 |
| Pi6 | P1 | 4.19 | 53074 | 47.86 |
| Pi7 | P1 | 3.28 | 40198 | 47.35 |
| Pi8 | P1 | 0.90 | 35251 | 16.35 |
| Pi100 | P10 | 4.05 | 54130 | 44.53 |
| Pi101 | P10 | 3.37 | 49895 | 41.38 |
| Pi102 | P10 | 4.76 | 51944 | 56.09 |
| Pi103 | P10 | 4.43 | 50714 | 59.17 |
| Pi105 | P10 | 5.10 | 57078 | 55.35 |
| Pi106 | P10 | 5.14 | 58513 | 54.78 |
| Pi97 | P10 | 3.97 | 57984 | 40.98 |
| Pi98 | P10 | 4.22 | 55307 | 45.7 |
| Pi99 | P10 | 3.78 | 55664 | 39.57 |
| Pi107 | P11 | 3.67 | 50641 | 43.94 |
| Pi108 | P11 | 4.83 | 53141 | 55.6 |
| Pi109 | P11 | 4.62 | 51075 | 55.45 |
| Pi110 | P11 | 2.88 | 37873 | 53.22 |
| Pi111 | P11 | 5.15 | 54060 | 58.56 |
| Pi112 | P11 | 3.84 | 50960 | 45.44 |
| Pi113 | P11 | 4.63 | 49659 | 58.83 |
| Pi114 | P11 | 2.70 | 59917 | 25.66 |
| Pi115 | P11 | 3.06 | 75941 | 23.58 |
| Pi116 | P11 | 3.07 | 58620 | 30.58 |
| Pi117 | P12 | 3.28 | 64649 | 29.29 |
| Pi118 | P12 | 3.79 | 74045 | 29.86 |
| Pi119 | P12 | 3.23 | 60860 | 32.17 |
| Pi120 | P12 | 3.83 | 57432 | 38.77 |
| Pi122 | P12 | 3.12 | 70601 | 25.65 |
| Pi123 | P12 | 2.57 | 76945 | 19.85 |
| Pi124 | P12 | 3.84 | 52510 | 48.14 |
| Pi125 | P12 | 4.07 | 61445 | 39.95 |
| Pi126 | P12 | 2.76 | 66023 | 22.66 |
| Pi127 | P12 | 1.78 | 52935 | 19.11 |
| Pi128 | P13 | 2.31 | 70658 | 20.04 |
| Pi129 | P13 | 2.06 | 59576 | 21.63 |
| Pi130 | P13 | 1.87 | 58341 | 19.23 |
| Pi131 | P13 | 1.10 | 44999 | 14.41 |
| Pi132 | P13 | 1.73 | 51963 | 20.35 |
| Pi133 | P13 | 3.05 | 64536 | 29.5 |
| Pi134 | P13 | 1.95 | 61767 | 19.09 |
| Pi135 | P13 | 1.62 | 42459 | 21.92 |
| Pi136 | P13 | 1.51 | 43954 | 21.47 |
| Pi137 | P13 | 4.25 | 72472 | 35.93 |
| Pi138 | P13 | 1.09 | 42400 | 16.63 |
| Pi140 | P13 | 0.98 | 51127 | 11.96 |
| Pi141 | P13 | 1.69 | 59883 | 16.58 |
| Pi142 | P13 | 1.94 | 59812 | 18.47 |
| Pi143 | P15 | 2.52 | 46081 | 32.29 |
| Pi144 | P15 | 1.99 | 36402 | 36.66 |
| Pi145 | P15 | 3.00 | 48941 | 40.53 |
| Pi146 | P15 | 2.77 | 59527 | 26.58 |
| Pi147 | P15 | 2.81 | 54106 | 31.55 |
| Pi149 | P15 | 3.80 | 56441 | 40.58 |
| Pi150 | P15 | 3.36 | 45121 | 46.22 |
| Pi151 | P15 | 3.14 | 44121 | 42.69 |
| Pi152 | P16 | 3.05 | 53490 | 33.63 |
| Pi153 | P16 | 1.89 | 77854 | 13.89 |
| Pi154 | P16 | 1.54 | 68074 | 12.54 |
| Pi155 | P16 | 1.57 | 40526 | 22.51 |
| Pi156 | P16 | 1.69 | 75672 | 12.01 |
| Pi157 | P16 | 2.56 | 50174 | 31.05 |
| Pi158 | P16 | 3.30 | 44822 | 44.21 |
| Pi159 | P16 | 3.24 | 48417 | 41.27 |
| Pi160 | P16 | 1.57 | 50122 | 19.68 |
| Pi11 | P2 | 4.83 | 59955 | 46.69 |
| Pi12 | P2 | 0.56 | 30029 | 12.09 |
| Pi13 | P2 | 4.51 | 52271 | 51.79 |
| Pi14 | P2 | 5.19 | 60401 | 52.56 |
| Pi15 | P2 | 3.75 | 73617 | 30.18 |
| Pi16 | P2 | 4.11 | 67446 | 35.34 |
| Pi17 | P2 | 4.45 | 67490 | 39.67 |
| Pi19 | P2 | 4.11 | 58180 | 40.65 |
| Pi20 | P2 | 4.89 | 61458 | 50.29 |
| Pi24 | P2 | 4.30 | 57376 | 46.37 |
| Pi25 | P2 | 4.33 | 58329 | 44.58 |
| Pi28 | P2 | 3.11 | 85348 | 22.06 |
| Pi30 | P3 | 0.78 | 35550 | 13.93 |
| Pi31 | P3 | 3.95 | 42133 | 60.81 |
| Pi32 | P3 | 5.53 | 69864 | 47.77 |
| Pi33 | P3 | 2.43 | 60237 | 24.09 |
| Pi34 | P3 | 3.76 | 67567 | 32.17 |
| Pi36 | P3 | 2.77 | 57117 | 30.32 |
| Pi37 | P3 | 3.36 | 58315 | 35.47 |
| Pi38 | P3 | 3.39 | 56365 | 37.67 |
| Pi39 | P3 | 2.22 | 49706 | 28.3 |
| Pi41 | P4 | 2.33 | 45783 | 33.05 |
| Pi42 | P4 | 2.39 | 51684 | 29.31 |
| Pi43 | P4 | 1.60 | 50395 | 20.14 |
| Pi45 | P4 | 4.47 | 57306 | 45.91 |
| Pi48 | P4 | 3.99 | 61304 | 39.1 |
| Pi50 | P4 | 3.53 | 56641 | 36.11 |
| Pi51 | P5 | 2.77 | 72476 | 23.52 |
| Pi52 | P5 | 2.93 | 73253 | 23.08 |
| Pi53 | P5 | 2.71 | 72245 | 22.74 |
| Pi54 | P5 | 3.44 | 82085 | 25.19 |
| Pi55 | P5 | 2.50 | 68367 | 21.87 |
| Pi56 | P5 | 2.62 | 64965 | 24.88 |
| Pi57 | P5 | 2.50 | 60398 | 24.02 |
| Pi58 | P5 | 2.45 | 64501 | 22.72 |
| Pi60 | P7 | 3.53 | 56159 | 39.48 |
| Pi61 | P7 | 3.70 | 55150 | 40.86 |
| Pi62 | P7 | 3.59 | 52178 | 41.2 |
| Pi63 | P7 | 4.24 | 58039 | 45.29 |
| Pi64 | P7 | 2.80 | 52837 | 33.39 |
| Pi65 | P7 | 3.38 | 48788 | 42.24 |
| Pi66 | P7 | 4.39 | 53702 | 48.53 |
| Pi67 | P7 | 1.85 | 59970 | 17.54 |
| Pi68 | P7 | 3.12 | 61603 | 29.88 |
| Pi69 | P7 | 2.35 | 65746 | 21.64 |
| Pi70 | P8 | 3.17 | 62297 | 29.89 |
| Pi71 | P8 | 3.34 | 66232 | 31.97 |
| Pi72 | P8 | 3.02 | 61021 | 29.61 |
| Pi73 | P8 | 0.75 | 28589 | 13.81 |
| Pi74 | P8 | 2.58 | 52224 | 29.26 |
| Pi75 | P8 | 3.10 | 54287 | 34.11 |
| Pi76 | P8 | 2.38 | 48459 | 29.28 |
| Pi77 | P8 | 2.03 | 48787 | 25.47 |
| Pi78 | P8 | 1.79 | 35402 | 33.28 |
| Pi79 | P8 | 3.80 | 69640 | 32.93 |
| Pi80 | P8 | 3.62 | 70128 | 29.73 |
| Pi81 | P8 | 4.39 | 70702 | 36.17 |
| Pi82 | P8 | 5.04 | 73269 | 39.74 |
| Pi83 | P8 | 4.12 | 67263 | 35.86 |
| Pi84 | P9 | 5.53 | 67447 | 48.06 |
| Pi85 | P9 | 4.52 | 69033 | 38.95 |
| Pi86 | P9 | 4.98 | 64526 | 44.89 |
| Pi87 | P9 | 4.48 | 69154 | 39.82 |
| Pi88 | P9 | 4.07 | 64184 | 36.84 |
| Pi89 | P9 | 4.46 | 64160 | 40.73 |
| Pi90 | P9 | 5.07 | 68794 | 43.58 |
| Pi91 | P9 | 3.72 | 60989 | 36.27 |
| Pi92 | P9 | 4.60 | 62838 | 44.08 |
| Pi93 | P9 | 2.19 | 48884 | 23.75 |
| Pi94 | P9 | 3.27 | 57154 | 35.65 |
| Pi95 | P9 | 4.55 | 57141 | 47.64 |
| Pi96 | P9 | 4.65 | 56879 | 49.01 |

**Table S5.** *Melanargia galathea* sequencing summary for individuals retained after individual-level missingness filtering, including number of reads, number of RAD loci and average sequencing depth per individual.

| Sample | Population | Reads (M) | Number of RAD loci | Average depth |
| --- | --- | --- | --- | --- |
| Mg35 | M10 | 2.803603 | 59380 | 21.43 |
| Mg36 | M10 | 2.557143 | 67999 | 16.86 |
| Mg37 | M10 | 2.62147 | 47455 | 22.19 |
| Mg38 | M10 | 3.032644 | 81624 | 16.79 |
| Mg39 | M10 | 3.539913 | 82385 | 19.93 |
| Mg40 | M10 | 2.869622 | 68173 | 19.07 |
| Mg41 | M10 | 3.328151 | 71595 | 20.75 |
| Mg42 | M10 | 4.696518 | 77805 | 27.05 |
| Mg43 | M10 | 4.299503 | 78417 | 24.31 |
| Mg44 | M11 | 4.6757 | 79081 | 28.07 |
| Mg45 | M11 | 3.923773 | 63720 | 27.36 |
| Mg46 | M11 | 4.199059 | 66768 | 28.4 |
| Mg47 | M11 | 4.063403 | 66725 | 26.72 |
| Mg48 | M11 | 3.830921 | 70069 | 25.75 |
| Mg49 | M11 | 3.251925 | 72704 | 20.58 |
| Mg50 | M11 | 3.791299 | 104938 | 16.17 |
| Mg51 | M11 | 3.978582 | 79921 | 22.68 |
| Mg52 | M11 | 3.884681 | 70875 | 23.64 |
| Mg53 | M12 | 2.943307 | 70492 | 19.39 |
| Mg54 | M12 | 3.975809 | 78697 | 24.01 |
| Mg55 | M12 | 3.119065 | 68759 | 21.47 |
| Mg56 | M12 | 3.477643 | 67146 | 22.32 |
| Mg57 | M12 | 3.776686 | 64311 | 26.67 |
| Mg58 | M12 | 3.536576 | 63315 | 25.72 |
| Mg59 | M12 | 3.794698 | 65712 | 26.78 |
| Mg60 | M12 | 3.747082 | 75578 | 22.95 |
| Mg61 | M12 | 4.513797 | 77194 | 26.51 |
| Mg62 | M12 | 3.13192 | 80785 | 17.96 |
| Mg63 | M12 | 3.039009 | 70151 | 20.78 |
| Mg64 | M15 | 2.802965 | 63389 | 19.83 |
| Mg65 | M15 | 2.645472 | 69282 | 18.56 |
| Mg66 | M15 | 3.49275 | 69302 | 24.05 |
| Mg67 | M15 | 2.645425 | 64046 | 19.28 |
| Mg68 | M15 | 2.843864 | 62354 | 21.57 |
| Mg69 | M15 | 3.790302 | 61817 | 28.76 |
| Mg70 | M15 | 3.573936 | 67857 | 25.66 |
| Mg71 | M15 | 3.223119 | 63998 | 23.6 |
| Mg72 | M15 | 2.548098 | 60002 | 19.37 |
| Mg73 | M15 | 2.504434 | 60455 | 18.71 |
| Mg74 | M16 | 4.214215 | 111563 | 16.6 |
| Mg75 | M16 | 3.634848 | 78979 | 21.57 |
| Mg76 | M16 | 3.921526 | 81881 | 21.88 |
| Mg77 | M16 | 0.879241 | 50713 | 8.26 |
| Mg78 | M16 | 5.751449 | 91514 | 28.88 |
| Mg79 | M16 | 5.191317 | 85700 | 27.63 |
| Mg80 | M16 | 5.442323 | 89410 | 28.22 |
| Mg81 | M16 | 5.186256 | 82936 | 30.35 |
| Mg82 | M16 | 5.646273 | 82599 | 30.49 |
| Mg10 | M7 | 3.651876 | 54179 | 30.78 |
| Mg11 | M7 | 2.234582 | 49596 | 20.82 |
| Mg12 | M7 | 2.178472 | 47472 | 19.75 |
| Mg13 | M7 | 1.536558 | 42613 | 15.95 |
| Mg14 | M7 | 1.86187 | 69131 | 11.7 |
| Mg15 | M7 | 2.211374 | 67576 | 15.95 |
| Mg6 | M7 | 3.026184 | 66703 | 20.85 |
| Mg7 | M7 | 2.208134 | 57591 | 17.05 |
| Mg8 | M7 | 1.453523 | 37702 | 14.92 |
| Mg9 | M7 | 3.062543 | 50963 | 27.09 |
| Mg16 | M8 | 0.87116 | 26496 | 11.77 |
| Mg17 | M8 | 1.606612 | 46078 | 14.42 |
| Mg19R | M8 | 3.949537 | 77580 | 23.56 |
| Mg20 | M8 | 3.030327 | 53566 | 25.64 |
| Mg21 | M8 | 2.157241 | 44266 | 19.8 |
| Mg22 | M8 | 3.016767 | 52062 | 25 |
| Mg23 | M8 | 4.047569 | 57212 | 30.92 |
| Mg24 | M9 | 2.96656 | 62315 | 21.44 |
| Mg25 | M9 | 2.415588 | 52838 | 20.53 |
| Mg26 | M9 | 2.454932 | 83724 | 13.38 |
| Mg27 | M9 | 2.423115 | 70849 | 16.25 |
| Mg28 | M9 | 2.527038 | 54789 | 20.09 |
| Mg29 | M9 | 3.503285 | 70800 | 23.44 |
| Mg30 | M9 | 3.767723 | 69471 | 25.34 |
| Mg31 | M9 | 3.754888 | 66430 | 24.98 |
| Mg32 | M9 | 4.218554 | 63067 | 29.03 |
| Mg33 | M9 | 3.979258 | 58401 | 29.89 |
| Mg34 | M9 | 3.39941 | 56286 | 24.32 |

**Table S6.** *Conocephalus fuscus* sequencing summary for individuals retained after individual-level missingness filtering, including number of reads, number of RAD loci and average sequencing depth per individual.

| Sample | Population | Reads (M) | Number of RAD loci | Average depth |
| --- | --- | --- | --- | --- |
| Cf1 | C1 | 4.458045 | 173376 | 16.32 |
| Cf2 | C1 | 4.894744 | 199157 | 15.39 |
| Cf4 | C1 | 5.078569 | 192314 | 16.11 |
| Cf5 | C1 | 4.458272 | 189082 | 14.1 |
| Cf6 | C1 | 4.939957 | 176393 | 17.12 |
| Cf7 | C1 | 4.133616 | 162369 | 15.83 |
| Cf8 | C1 | 3.14432 | 142081 | 14.2 |
| Cf9 | C1 | 4.715561 | 184367 | 15.87 |
| Cf100 | C11 | 5.514277 | 190831 | 19.01 |
| Cf101 | C11 | 4.763978 | 167442 | 18.73 |
| Cf102 | C11 | 5.485154 | 184166 | 19.5 |
| Cf103 | C11 | 5.959114 | 192894 | 19.56 |
| Cf105 | C11 | 6.042171 | 202394 | 19.61 |
| Cf95 | C11 | 5.538932 | 199286 | 17.27 |
| Cf96 | C11 | 4.779316 | 197826 | 15.41 |
| Cf97 | C11 | 4.788074 | 183469 | 16.47 |
| Cf98 | C11 | 5.161698 | 190696 | 17.18 |
| Cf99 | C11 | 5.081205 | 195107 | 16.73 |
| Cf106 | C12 | 4.352339 | 164889 | 18.01 |
| Cf107 | C12 | 6.164629 | 220248 | 18.22 |
| Cf108 | C12 | 2.593312 | 117947 | 15.23 |
| Cf109 | C12 | 4.028423 | 143783 | 18.7 |
| Cf110 | C12 | 5.032568 | 190188 | 17.55 |
| Cf111 | C12 | 5.462029 | 209574 | 16.53 |
| Cf112 | C12 | 6.053498 | 207207 | 18.54 |
| Cf113 | C12 | 5.344767 | 152805 | 23.07 |
| Cf114 | C12 | 5.580145 | 158828 | 23.39 |
| Cf115 | C12 | 6.782506 | 216374 | 19.31 |
| Cf116 | C12 | 5.259333 | 185832 | 18.39 |
| Cf117 | C13 | 6.057226 | 210227 | 18.44 |
| Cf118R | C13 | 4.622279 | 164292 | 17.49 |
| Cf119 | C13 | 6.073406 | 201962 | 19.33 |
| Cf120 | C13 | 5.003873 | 179769 | 17.69 |
| Cf121 | C13 | 5.479541 | 205640 | 17.24 |
| Cf122 | C14 | 4.946957 | 181159 | 17.68 |
| Cf123 | C14 | 4.828689 | 189507 | 16.94 |
| Cf124 | C14 | 4.551659 | 197231 | 15.08 |
| Cf125 | C14 | 4.284699 | 165280 | 17.36 |
| Cf126 | C14 | 4.711473 | 166446 | 18.93 |
| Cf127 | C14 | 5.319334 | 202682 | 17.13 |
| Cf128 | C14 | 4.387021 | 179796 | 15.99 |
| Cf129 | C14 | 6.070646 | 186383 | 21.79 |
| Cf130 | C14 | 4.413993 | 172752 | 17.28 |
| Cf131 | C14 | 5.819685 | 214500 | 18.17 |
| Cf132 | C15 | 5.070969 | 174071 | 19.07 |
| Cf133 | C15 | 5.080457 | 199083 | 16.43 |
| Cf134 | C15 | 5.193289 | 190826 | 18.31 |
| Cf135 | C15 | 5.743236 | 219125 | 16.78 |
| Cf136 | C15 | 4.630054 | 209702 | 14.21 |
| Cf137 | C15 | 5.339632 | 207465 | 16.08 |
| Cf138 | C15 | 5.794332 | 216905 | 16.75 |
| Cf139 | C15 | 6.779007 | 249137 | 16.98 |
| Cf140 | C15 | 5.539096 | 216376 | 16.43 |
| Cf141 | C15 | 4.921177 | 209113 | 15.38 |
| Cf142 | C16 | 4.745653 | 186342 | 16.75 |
| Cf143 | C16 | 5.889469 | 222255 | 16.92 |
| Cf144 | C16 | 5.065614 | 198476 | 16.89 |
| Cf145 | C16 | 5.110254 | 222607 | 14.89 |
| Cf146 | C16 | 4.655288 | 184358 | 16.33 |
| Cf147 | C16 | 4.910442 | 165509 | 18.66 |
| Cf148 | C16 | 4.751734 | 163441 | 18.08 |
| Cf149 | C16 | 4.975057 | 165411 | 18.82 |
| Cf150 | C16 | 4.302506 | 150643 | 18.13 |
| Cf151 | C16 | 5.627399 | 186440 | 18.74 |
| Cf152 | C16 | 4.631832 | 157916 | 18.26 |
| Cf153 | C16 | 5.525716 | 175760 | 19.67 |
| Cf14 | C2 | 5.109668 | 189295 | 17.12 |
| Cf15 | C2 | 4.638813 | 192428 | 14.53 |
| Cf16 | C2 | 3.86392 | 184861 | 12.65 |
| Cf17 | C2 | 4.048146 | 197764 | 12.66 |
| Cf18 | C2 | 4.660291 | 198980 | 14.02 |
| Cf19 | C2 | 4.913032 | 197894 | 14.92 |
| Cf20 | C2 | 4.698549 | 203612 | 14.19 |
| Cf21 | C2 | 5.227656 | 200864 | 16.09 |
| Cf22 | C2 | 4.274841 | 181727 | 14.92 |
| Cf23 | C2 | 5.440609 | 214017 | 15.4 |
| Cf24 | C2 | 4.678282 | 199635 | 14.17 |
| Cf25 | C2 | 2.229894 | 135766 | 10.27 |
| Cf26 | C2 | 4.909185 | 215894 | 14.16 |
| Cf27 | C2 | 5.025438 | 213591 | 14.35 |
| Cf28 | C3 | 4.374306 | 197559 | 13.65 |
| Cf29 | C3 | 4.738991 | 210326 | 13.94 |
| Cf30 | C3 | 2.053529 | 128878 | 10.15 |
| Cf31 | C4 | 4.826952 | 199693 | 14.95 |
| Cf32 | C4 | 4.87764 | 210819 | 13.99 |
| Cf33 | C4 | 4.839014 | 198942 | 15.41 |
| Cf34 | C4 | 3.207587 | 155898 | 12.98 |
| Cf35 | C4 | 4.942255 | 192043 | 16.27 |
| Cf36 | C4 | 3.235167 | 171835 | 11.74 |
| Cf37 | C4 | 3.534876 | 185185 | 11.93 |
| Cf38 | C4 | 4.156894 | 198131 | 12.86 |
| Cf39 | C4 | 5.555556 | 213073 | 15.72 |
| Cf40 | C5 | 3.830622 | 194680 | 12.16 |
| Cf41 | C5 | 4.224083 | 210200 | 12.08 |
| Cf42 | C5 | 4.844069 | 216897 | 13.51 |
| Cf43 | C5 | 4.336412 | 210908 | 12.08 |
| Cf44 | C5 | 4.465957 | 204838 | 12.71 |
| Cf45 | C5 | 4.232958 | 191992 | 13.74 |
| Cf46 | C5 | 4.190491 | 194248 | 13.05 |
| Cf47 | C5 | 5.44338 | 214510 | 15.02 |
| Cf48 | C5 | 4.466829 | 216865 | 12.34 |
| Cf49 | C5 | 4.587943 | 200891 | 13.67 |
| Cf50 | C5 | 4.338603 | 198587 | 13.65 |
| Cf51 | C5 | 4.779391 | 229779 | 13.01 |
| Cf52 | C6 | 5.071707 | 190538 | 16.27 |
| Cf53 | C6 | 5.138384 | 195841 | 15.89 |
| Cf54 | C6 | 5.432631 | 201791 | 16.42 |
| Cf55 | C6 | 5.000279 | 191484 | 15.51 |
| Cf56 | C6 | 5.028113 | 191111 | 15.81 |
| Cf57 | C6 | 4.185995 | 169134 | 15.43 |
| Cf58 | C6 | 4.559566 | 179012 | 15.83 |
| Cf59 | C6 | 4.635482 | 181516 | 15.67 |
| Cf60 | C6 | 4.914229 | 192480 | 16.03 |
| Cf61 | C6 | 4.484674 | 185323 | 14.86 |
| Cf62 | C6 | 4.213942 | 185805 | 13.87 |
| Cf63 | C6 | 4.718299 | 202188 | 14.05 |
| Cf64 | C7 | 4.187905 | 207774 | 12.29 |
| Cf65 | C7 | 5.653835 | 242820 | 13.94 |
| Cf66 | C7 | 5.304274 | 228913 | 13.59 |
| Cf67 | C7 | 5.066439 | 226994 | 13.27 |
| Cf68 | C7 | 4.521613 | 217153 | 12.61 |
| Cf69 | C7 | 4.860698 | 213606 | 14.03 |
| Cf70 | C7 | 4.754757 | 215873 | 13.25 |
| Cf71 | C7 | 5.684719 | 230592 | 14.69 |
| Cf72 | C7 | 6.343944 | 247079 | 15.16 |
| Cf73 | C7 | 4.9741 | 219382 | 13.3 |
| Cf74 | C8 | 4.403425 | 211767 | 12.38 |
| Cf75 | C8 | 4.764609 | 241845 | 11.79 |
| Cf76 | C8 | 4.457356 | 208411 | 13.31 |
| Cf77 | C8 | 5.076571 | 230200 | 13.72 |
| Cf78 | C8 | 5.281763 | 245550 | 13.09 |
| Cf79 | C8 | 5.418872 | 236826 | 13.75 |
| Cf80 | C8 | 5.094182 | 234559 | 13.44 |
| Cf81 | C8 | 5.065837 | 209680 | 15.15 |
| Cf82 | C8 | 4.99053 | 205950 | 15.21 |
| Cf83 | C8 | 5.6309 | 222842 | 15.81 |
| Cf84 | C9 | 5.538465 | 232180 | 14.34 |
| Cf85 | C9 | 4.922842 | 219199 | 13.94 |
| Cf86 | C9 | 4.997571 | 232296 | 13.31 |
| Cf87 | C9 | 5.437632 | 223122 | 14.46 |
| Cf88 | C9 | 4.698223 | 181409 | 16.33 |
| Cf89 | C9 | 5.724355 | 214362 | 16.57 |
| Cf90 | C9 | 6.271698 | 227438 | 16.71 |
| Cf91 | C9 | 6.294294 | 215762 | 17.76 |
| Cf92 | C9 | 5.80382 | 181858 | 20.09 |
| Cf93 | C9 | 4.858569 | 191209 | 16.28 |
| Cf94 | C9 | 4.755189 | 190148 | 15.77 |

**Table S7.** Number of genomic sites retained during preparation of the analysis-specific datasets for each species. The genome-wide genetic diversity dataset contained both polymorphic and monomorphic sites, whereas the dataset used for genetic differentiation and population-structure analyses contained only polymorphic SNPs. For *P. icarus* and *M. galathea*, the reported numbers correspond to datasets after exclusion of sex-linked sites. Counts are reported for filtering stages for which retained-site numbers were recorded.

| **Filtering step / analytical dataset** | ***P. icarus*** | ***M. galathea*** | ***C. fuscus*** |
| --- | --- | --- | --- |
| Initial *ipyrad* output | 14,104,825 | 8,074,859 | 20,608,081 |
| After --max-missing 0.85 | 501,542 | 1,149,641 | 1,081,825 |
| — Monomorphic sites | 398,363 | 1,104,760 | 1,025,424 |
| — Polymorphic sites | 103,179 | 44,881 | 56,401 |
| **Genome-wide genetic diversity dataset** | **501,542** | **1,149,641** | **1,081,825** |
| After --maf 0.05¹ | 6,642 | 22,493 | 13,160 |
| After LD pruning¹ | 5,038 | 15,561 | 9,678 |
| **Final SNP dataset** | **5,038** | **15,561** | **9,678** |

¹ Applied only to the polymorphic-site dataset.

**Table S8.** Correlation coefficients between landscape predictors and genome-wide genetic diversity (uHEXP) for the three focal species. Highlighted values indicate the spatial radius with the largest absolute correlation coefficient for each predictor and species.

|  | *Polyommatus icarus* *uH*_EXP_ | *Melanargia galathea* *uH*_EXP_ | *Conocephalus fuscus* *uH*_EXP_ |
| --- | --- | --- | --- |
| Edge density 500m | 0.27 | 0.63 | 0.67 |
| Edge density 700m | 0.15 | 0.49 | 0.65 |
| Edge density 1000m | 0.09 | 0.40 | 0.63 |
|  |  |  |  |
| Fragmentation of extensive meadows 500m | -0.34 | -0.09 | 0.17 |
| Fragmentation of extensive meadows 700m | -0.18 | -0.33 | 0.05 |
| Fragmentation of extensive meadows 1000m | -0.13 | -0.39 | 0.32 |
|  |  |  |  |
| Proportion of arable 500m | -0.41 | -0.26 | 0.07 |
| Proportion of arable 700m | -0.47 | -0.43 | 0.11 |
| Proportion of arable 1000m | -0.39 | -0.42 | 0.12 |
|  |  |  |  |
| Proportion of extensive meadows 500m | 0.17 | 0.34 | 0.20 |
| Proportion of extensive meadows 700m | 0.18 | 0.38 | 0.16 |
| Proportion of extensive meadows 1000m | 0.24 | 0.48 | 0.06 |
|  |  |  |  |
| Proportion of forest 500m | 0.10 | -0.11 | -0.28 |
| Proportion of forest 700m | 0.15 | -0.09 | -0.25 |
| Proportion of forest 1000m | 0.16 | -0.17 | -0.25 |
|  |  |  |  |
| Proportion of intensive meadows 500m | -0.12 | -0.13 | -0.14 |
| Proportion of intensive meadows 700m | -0.08 | -0.12 | -0.18 |
| Proportion of intensive meadows 1000m | -0.06 | -0.17 | -0.13 |

**Table S9.** *Polyommatus icarus* estimated pairwise *F*_ST_ between all location pairs (below diagonal). P-values from 1000 bootstrap replicates (above diagonal).

|  | P10 | P11 | P12 | P2 | P13 | P15 | P16 | P1 | P3 | P4 | P5 | P7 | P8 | P9 |
| --- | --- | --- | --- | --- | --- | --- | --- | --- | --- | --- | --- | --- | --- | --- |
| P10 | NA | 0.04 | 0 | 0 | 0 | 0 | 0.11 | 0.05 | 0.02 | 0 | 0 | 0.01 | 0.01 | 0 |
| P11 | 0.002918 | NA | 0 | 0.09 | 0.09 | 0.22 | 0.98 | 0.38 | 0.37 | 0.04 | 0 | 0.18 | 0.59 | 0.03 |
| P12 | 0.007946 | 0.006228 | NA | 0 | 0 | 0.03 | 0.02 | 0 | 0 | 0 | 0 | 0 | 0 | 0 |
| P2 | 0.003946 | 0.001515 | 0.006466 | NA | 0.91 | 0.08 | 0.92 | 0.18 | 0.73 | 0.06 | 0 | 0.39 | 0.72 | 0.06 |
| P13 | 0.003723 | 0.001398 | 0.007053 | 0 | NA | 0.09 | 0.55 | 0.03 | 0.58 | 0 | 0 | 0.04 | 0.57 | 0.13 |
| P15 | 0.006047 | 0.001424 | 0.004553 | 0.00299 | 0.002266 | NA | 0.42 | 0.24 | 0.13 | 0.18 | 0 | 0.18 | 0.03 | 0.02 |
| P16 | 0.002915 | 0 | 0.003249 | 0 | 0 | 0.000106 | NA | 0.56 | 0.86 | 0.07 | 0 | 0.95 | 0.57 | 0.48 |
| P1 | 0.003109 | 0.000676 | 0.007403 | 0.001972 | 0.003984 | 0.001631 | 0 | NA | 0.24 | 0 | 0 | 0.34 | 0.13 | 0 |
| P3 | 0.004379 | 0.000286 | 0.004318 | 0 | 0.00E+00 | 0.002523 | 0 | 0.001837 | NA | 0.07 | 0 | 0.17 | 0.64 | 0.05 |
| P4 | 0.006216 | 0.004091 | 0.006518 | 0.003326 | 0.007679 | 0.002783 | 0.004465 | 0.009498 | 0.003798 | NA | 0 | 0.02 | 0.01 | 0.02 |
| P5 | 0.010527 | 0.006549 | 0.010331 | 0.007755 | 0.00452 | 0.007626 | 0.008378 | 0.012858 | 0.005853 | 0.013289 | NA | 0 | 0 | 0 |
| P7 | 0.005595 | 0.001318 | 0.007405 | 0.000915 | 0.002652 | 0.001978 | 0 | 0.001123 | 0.00192 | 0.00451 | 0.006779 | NA | 0.18 | 0.01 |
| P8 | 0.003433 | 0 | 0.007431 | 0 | 0 | 0.002937 | 0 | 0.002261 | 0 | 0.005178 | 0.007618 | 0.001497 | NA | 0.01 |
| P9 | 0.004934 | 0.002274 | 0.007736 | 0.002104 | 0.001587 | 0.003852 | 0 | 0.004659 | 0.002556 | 0.004586 | 0.006175 | 0.003578 | 0.003005 | NA |

**Table S10.** *Melanargia galathea* estimated pairwise *F*_ST_ between all location pairs (below diagonal). P-values from 1000 bootstrap replicates (above diagonal).

|  | M7 | M8 | M9 | M10 | M11 | M12 | M15 | M16 |
| --- | --- | --- | --- | --- | --- | --- | --- | --- |
| M7 | NA | 0 | 0 | 0 | 0 | 0 | 0 | 0 |
| M8 | 0.005279 | NA | 0 | 0 | 0 | 0 | 0 | 0 |
| M9 | 0.011915 | 0.008547 | NA | 0 | 0 | 0 | 0 | 0 |
| M10 | 0.013142 | 0.009042 | 0.009871 | NA | 0 | 0 | 0 | 0 |
| M11 | 0.010773 | 0.007977 | 0.007386 | 0.01095 | NA | 0 | 0 | 0 |
| M12 | 0.010405 | 0.006242 | 0.008322 | 0.010753 | 0.005237 | NA | 0 | 0 |
| M15 | 0.038461 | 0.036318 | 0.035334 | 0.038813 | 0.036488 | 0.035864 | NA | 0 |
| M16 | 0.016484 | 0.012318 | 0.015692 | 0.015449 | 0.013103 | 0.012315 | 0.035425 | NA |

**Table S11.** *Conocephalus fuscus* estimated pairwise *F*_ST_ between all location pairs (below diagonal). P-values from 1000 bootstrap replicates (above diagonal).

|  | C11 | C12 | C13 | C14 | C15 | C16 | C2 | C1 | C3 | C4 | C5 | C6 | C7 | C8 | C9 |
| --- | --- | --- | --- | --- | --- | --- | --- | --- | --- | --- | --- | --- | --- | --- | --- |
| C11 | NA | 0 | 0 | 0 | 0 | 0 | 0 | 0 | 0 | 0 | 0 | 0 | 0 | 0 | 0 |
| C12 | 0.0632 | NA | 0 | 0 | 0 | 0 | 0 | 0 | 0 | 0 | 0 | 0 | 0 | 0 | 0 |
| C13 | 0.0428 | 0.0470 | NA | 0 | 0 | 0 | 0 | 0 | 0.46 | 0 | 0 | 0 | 0 | 0 | 0 |
| C14 | 0.0500 | 0.0639 | 0.0262 | NA | 0 | 0 | 0 | 0 | 0 | 0 | 0 | 0 | 0 | 0 | 0 |
| C15 | 0.0902 | 0.0987 | 0.0607 | 0.0856 | NA | 0 | 0 | 0 | 0 | 0 | 0 | 0 | 0 | 0 | 0 |
| C16 | 0.0836 | 0.0976 | 0.0765 | 0.0811 | 0.1180 | NA | 0 | 0 | 0 | 0 | 0 | 0 | 0 | 0 | 0 |
| C2 | 0.0565 | 0.0720 | 0.0534 | 0.0491 | 0.1138 | 0.0922 | NA | 0 | 0 | 0 | 0 | 0 | 0 | 0 | 0 |
| C1 | 0.0616 | 0.0764 | 0.0565 | 0.0559 | 0.1288 | 0.1000 | 0.0394 | NA | 0 | 0 | 0 | 0 | 0 | 0 | 0 |
| C3 | 0.0232 | 0.0365 | 0.0013 | 0.0109 | 0.0850 | 0.0686 | 0.0338 | 0.0358 | NA | 0 | 0 | 0 | 0 | 0 | 0 |
| C4 | 0.0430 | 0.0525 | 0.0237 | 0.0393 | 0.0717 | 0.0787 | 0.0540 | 0.0555 | 0.0155 | NA | 0 | 0 | 0 | 0 | 0 |
| C5 | 0.0363 | 0.0490 | 0.0206 | 0.0384 | 0.0646 | 0.0723 | 0.0512 | 0.0572 | 0.0155 | 0.0224 | NA | 0 | 0 | 0 | 0 |
| C6 | 0.0859 | 0.0952 | 0.0803 | 0.0844 | 0.1288 | 0.1129 | 0.0872 | 0.0937 | 0.0677 | 0.0752 | 0.0725 | NA | 0 | 0 | 0 |
| C7 | 0.0621 | 0.0730 | 0.0494 | 0.0591 | 0.0922 | 0.0993 | 0.0712 | 0.0831 | 0.0468 | 0.0502 | 0.0430 | 0.0985 | NA | 0 | 0 |
| C8 | 0.0931 | 0.1067 | 0.0743 | 0.0928 | 0.1155 | 0.1246 | 0.1079 | 0.1196 | 0.0816 | 0.0797 | 0.0701 | 0.1276 | 0.0970 | NA | 0 |
| C9 | 0.0595 | 0.0712 | 0.0461 | 0.0609 | 0.0820 | 0.0900 | 0.0739 | 0.0824 | 0.0463 | 0.0509 | 0.0431 | 0.0965 | 0.0678 | 0.0987 | NA |
